# Generalised Geometric Brownian Motion: Theory and Applications to Option Pricing

**DOI:** 10.3390/e22121432

**Published:** 2020-12-18

**Authors:** Viktor Stojkoski, Trifce Sandev, Lasko Basnarkov, Ljupco Kocarev, Ralf Metzler

**Affiliations:** 1 Faculty of Economics, Ss. Cyril and Methodius University, 1000 Skopje, Macedonia; vstojkoski@manu.edu.mk; 2 Research Centre for Computer Science and Information Technologies, Macedonian Academy of Sciences and Arts, Bul. Krste Misirkov 2, 1000 Skopje, Macedonia; trifce.sandev@manu.edu.mk (T.S.); lasko.basnarkov@finki.ukim.mk (L.B.); lkocarev@manu.edu.mk (L.K.); 3 Institute of Physics & Astronomy, University of Potsdam, D-14776 Potsdam-Golm, Germany; 4 Institute of Physics, Faculty of Natural Sciences and Mathematics, Ss. Cyril and Methodius University, Arhimedova 3, 1000 Skopje, Macedonia; 5 Faculty of Computer Science and Engineering, Ss. Cyril and Methodius University, P.O. Box 393, 1000 Skopje, Macedonia

**Keywords:** geometric Brownian motion, Fokker–Planck equation, Black–Scholes model, option pricing

## Abstract

Classical option pricing schemes assume that the value of a financial asset follows a geometric Brownian motion (GBM). However, a growing body of studies suggest that a simple GBM trajectory is not an adequate representation for asset dynamics, due to irregularities found when comparing its properties with empirical distributions. As a solution, we investigate a generalisation of GBM where the introduction of a memory kernel critically determines the behaviour of the stochastic process. We find the general expressions for the moments, log-moments, and the expectation of the periodic log returns, and then obtain the corresponding probability density functions using the subordination approach. Particularly, we consider subdiffusive GBM (sGBM), tempered sGBM, a mix of GBM and sGBM, and a mix of sGBMs. We utilise the resulting generalised GBM (gGBM) in order to examine the empirical performance of a selected group of kernels in the pricing of European call options. Our results indicate that the performance of a kernel ultimately depends on the maturity of the option and its moneyness.

## 1. Introduction

Geometric Brownian motion (GBM) frequently features in mathematical modelling. The advantage of modelling through this process lies in its universality, as it represents an attractor of more complex models that exhibit non-ergodic dynamics [1,2,3]. As such, GBM has been used to underlie the dynamics of a diverse set of natural phenomena, including the distribution of incomes, body weights, rainfall, fragment sizes in rock crushing processes, etc. [4,5]. Nevertheless, perhaps the best-known application of GBM is in finance, and, in particular, in terms of the Black–Scholes (BS) model (or Black–Scholes–Merton model) [6,7,8] for the pricing of European options.

By construction, GBM is a simple continuous-time stochastic process in which the logarithm of the randomly varying quantity of interest follows a Brownian motion with drift. Its non-ergodicity is manifested in the difference between the growth rate that was observed in an individual trajectory and the ensemble average growth [9]. The time-averaged growth rate is dependent on both the drift and randomness in the system, whereas the ensemble growth rate is solely dependent on the drift. If only a single system is to be modelled, in the long run only the time-averaged growth rate is observed. This is naturally the case in financial market dynamics, for which only single time series exist, and where individual realisations would be expected to be distinctly disparate [10].

Moreover, GBM is closely related to the problem of heterogeneous diffusion and turbulent diffusion, which are represented by the inhomogeneous advection–diffusion equation with position-dependent diffusion coefficient D(x) and velocity field v(x). It is well known that, at a turbulent diffusion, the contaminant spreads very fast. For the case of Richardson diffusion, the position-dependent diffusion coefficient behaves as $D\left(x\right)∼x^{4/3}$ and the relative mean squared displacement (MSD) scales as $\langle x^{2}\left(t\right)\rangle ∼t^{3}$ [11]. However, the fast spread of contaminants can be essentially increased due to multiplicative noise, such that the MSD grows exponentially with time [12,13].

Notably, in a variety of cases, GBM has failed to reproduce the properties of real asset prices. For instance, by definition, GBM is not able to adequately reproduce fat-tailed distributions of various characteristics observed in reality [14]. As a solution, several alternating theories have been proposed, among which are: stochastic volatility [15,16,17]; local volatility [18,19]; time-varying volatility [20,21], models utilising stochastic processes in which the noise follows a fat-tailed distribution [22,23,24,25,26,27]; and, generalisations of GBM based on subdiffusion [28,29,30]. In the first approach, the volatility is a stochastic process itself. In local volatility models, asset prices follow a stochastic differential equation whose diffusion coefficient is a function of the price. The time-varying volatility models assume that the latent volatility is predictable with respect to the information set. In contrast to stochastic volatility, in these models the conditional variance is a deterministic function of the model parameters and past data. Utilising stochastic processes in which the noise follows a fat-tailed distribution intuitively leads to the desired result. However, it has been acknowledged that several models that are described as a fat-tailed process can, in truth, be derived from stochastic and local volatility models [31,32,33,34]. The last, i.e., the subdiffusive approach, differently from the other views, assumes anomalous price dynamics. Concretely, the observation that the distribution of log returns is fat-tailed can be attributed to prolonged periods, in which the price of the asset exhibits approximately constant extreme values. These constant periods can be considered to be trapping of particles, as is done in physical systems that manifest anomalous diffusion (subdiffusion) [35,36]. While the resulting subdiffusive GBM (sGBM) is able to easily reproduce real-life properties, the literature lacks an extensive study in which the exact empirical characteristics of the subdiffusive model are presented.

The purpose of this paper is to propose a unifying framework for the application of subdiffusive GBM models in option pricing. We do this by providing a thorough investigation on the properties of the so-called generalised GBM (gGBM) [37]. gGBM is a stochastic process whose behaviour is critically determined by a memory kernel. By choosing the appropriate kernel, we recover the standard GBM and the typically used subdiffusive GBM models [28,29,30]. In order to understand the behaviour of gGBM under various kernels, we perform a detailed analysis of the moments, log-moments, and the expectation of the periodic log returns, and obtain the corresponding probability density functions by using the subordination approach. We show that the dynamics of the model can be easily adjusted in order to mimic periods of constant prices and/or fat-tailed observations of returns, thus corresponding to realistic scenarios. More importantly, it is known that gGBM leads to risk-neutral asset price dynamics, and, thus, it is adequate for their modelling [37]. We utilise this property of the model to investigate its capability to predict empirical option values. We find that the performance of a kernel ultimately depends on the parameters of the option, such as its maturity and moneyness. The first property describes the time that is left for the option to be exercised, whereas the second characteristic depicts the relative position of the current price with respect to the strike price of the option. At first sight, this conclusion appears intuitive—obviously the known information for the properties of the asset greatly impacts its price. However, the observation that a slight change in the known information may drastically change the dynamics suggests that there is a need in the option pricing literature for models that easily allow for such structural changes. We believe that the resolution to this issue lies in applying the concepts of time-averaging and ergodicity breaking to modelling financial time-series, and the gGBM framework offers a computationally inexpensive and efficiently tractable solution.

The paper is organised as follows. In Section 2, we provide an overview of GBM in the BS model and its use in option pricing. We also give detailed results for the so-called sGBM in terms of the fractional Fokker–Planck equation and its corresponding continuous time random walk (CTRW) model. In Section 3, we present gGBM and describe its properties using the subordination approach. In particular, we derive the corresponding Fokker–Planck equation with a memory kernel and obtain the respective moments and log-moments. The general function that is used in the Lévy exponent occurs as a memory kernel in the Fokker–Planck equation, which allows for us to recover the previously known results for GBM and sGBM. We consider generalisations of GBM and sGBM by introducing tempered sGBM, a mix of GBM and sGBM, as well as a mix of sGBMs. A numerical investigation of the properties of the model is given in Section 4 and an empirical example of application of the gGBM in option pricing is presented in Section 5. Section 6 summarises our findings. In the Appendices, we give detailed calculations as well as derivation of the Fokker–Planck equation for the gGBM within the CTRW theory.

## 2. Background

### 2.1. Standard GBM

GBM has been applied in a variety of scientific fields [1,3,9,38,39,40]. Mathematically, it is represented by the Langevin equation

(1) $$\begin{array}{c} dx\left(t\right)=x\left(t\right)\left[\mu \;dt+\sigma \;dB(t)\right],\;x_{0}=x\left(0\right), \end{array}$$

where x(t) is the particle position, μ is the drift, σ>0 is the volatility, and B(t) represents a standard Brownian motion. The solution to Equation (1), in the Itô sense, is

(2) $$\begin{array}{c} x\left(t\right)=x_{0}\;e^{\left(\mu -\frac{\sigma^{2}}{2}\right)t+\sigma B\left(t\right)},\;x_{0}=x\left(0\right)>0. \end{array}$$

When the dynamics of the asset price follows a GBM, then a risk-neutral distribution (probability distribution that takes into account the risk of future price fluctuations) can be easily found by solving the corresponding Fokker–Planck equation to Equation (1),

(3) $$\begin{array}{c} \frac{\partial }{\partial t}f\left(x,t\right)=-\mu \frac{\partial }{\partial x}xf\left(x,t\right)+\frac{\sigma^{2}}{2}\frac{\partial^{2}}{\partial x^{2}}x^{2}f\left(x,t\right), \end{array}$$

with initial condition $f\left(x,t=0\right)=\delta \left(x-x_{0}\right)$. The solution of Equation (3) is the famed log-normal distribution

(4) $$\begin{array}{c} f\left(x,t\right)=\frac{1}{x\sqrt{2\pi \sigma^{2}t}}\times \exp\left(-\frac{{\left[\log x-\log x_{0}-\bar{\mu }\;t\right]}^{2}}{2\sigma^{2}t}\right). \end{array}$$

where $\bar{\mu }=\mu -\sigma^{2}/2$. We point out that this representation corresponds to the Itô interpretation of the multiplicative noise. There are also Stratonovich and Klimontovich–Hänggi interpretations, for which the corresponding Fokker–Planck equations are slightly different, see Refs. [12,41]. In finance math literature, the Itô convention is the standard interpretation.

From the solution, it follows that the mean value and mean squared displacement (MSD) have exponential dependence on time,

(5) $$\begin{array}{c} \langle x\left(t\right)\rangle =x_{0}\;e^{\mu \;t}, \end{array}$$

and

(6) $$\begin{array}{c} \langle x^{2}\left(t\right)\rangle =x_{0}^{2}\;e^{(\sigma^{2}+2\;\mu )t}, \end{array}$$

respectively. Thus, the variance becomes

(7) $$\begin{array}{c} \langle x^{2}\left(t\right)\rangle -{\langle x(t)\rangle }^{2}=x_{0}^{2}\;e^{2\mu t}\left(e^{\sigma^{2}t}-1\right). \end{array}$$

The exact derivation of the GBM distribution and its moments is given in Appendix A. In the same way, one calculates the third and fourth moments, which are given by

(8) $$\begin{array}{c} \langle x^{3}\left(t\right)\rangle =x_{0}^{3}\;e^{(3\sigma^{2}+3\mu )t}, \end{array}$$

(9) $$\begin{array}{c} \langle x^{4}\left(t\right)\rangle =x_{0}^{4}\;e^{(6\sigma^{2}+4\mu )t}, \end{array}$$

respectively. The third and fourth moments are used to estimate the skewness *g*, and, respectively, excess kurtosis κ of the probability distribution of a random variable *y*,

(10) $$\begin{array}{cc} g & =\frac{\langle {\left(y-\langle y\rangle \right)}^{3}\rangle }{{\left(\langle y^{2}\rangle -{\langle y\rangle }^{2}\right)}^{3/2}}, \end{array}$$

(11) $$\begin{array}{cc} \kappa & =\frac{\langle {\left(y-\langle y\rangle \right)}^{4}\rangle }{{\left(\langle y^{2}\rangle -{\langle y\rangle }^{2}\right)}^{2}}-3. \end{array}$$

The skewness is a measure of the asymmetry of the probability distribution of a real-valued random variable around its first moment, whereas the excess kurtosis evaluates the “tailedness” of the probability distribution. From these relations for the random variable *x*, one finds the skewness and excess kurtosis in the form

(12) $$\begin{array}{c} g=\sqrt{e^{\sigma^{2}t}-1}\;\left(e^{\sigma^{2}t}+2\right), \end{array}$$

(13) $$\begin{array}{c} \kappa =e^{4\sigma^{2}t}+2e^{3\sigma^{2}t}+3e^{2\sigma^{2}t}-6. \end{array}$$

Evidently, in GBM, the diffusion coefficient scales proportionally with the square of the position of the particle, i.e., $D\left(x\right)=\sigma^{2}x^{2}/2$, and, thus, the MSD has an exponential dependence on time. A more convenient measure instead of the MSD for geometric processes is the behaviour of the expectation of the logarithm of x(t), which, in asset pricing terms, represents the continuously compounded return of the asset. In the case of GBM, the expectation of the logarithm of the particle position has a linear dependence on time. This can be shown by calculation of the log-moments $\langle \log^{n}x(t)\rangle =\int_{0}^{\infty }\log^{n}x\;P\left(x,t\right)\;dx$, see Appendix A, Equation (A7). The mean value of the logarithm of x(t) becomes

(14) $$\begin{array}{c} \langle \log x(t)\rangle =\langle \log x_{0}\rangle +\bar{\mu }\;t, \end{array}$$

from where for the expectation of the periodic log return with period Δt, one finds

(15) $$\begin{array}{c} \frac{1}{\Delta t}\langle \log\left(x(t+\Delta t)/x(t)\right)\rangle \underset{\Delta t\to 0}{∼}\bar{\mu }=\frac{d}{dt}\langle \log x(t)\rangle . \end{array}$$

The second log-moment is given by

(16) $$\begin{array}{c} \langle \log^{2}x(t)\rangle =\langle \log^{2}x_{0}\rangle +\left\{2\bar{\mu }\langle \log x_{0}\rangle +\sigma^{2}\right\}t+{\bar{\mu }}^{2}t^{2}, \end{array}$$

which for the log-variance yields

(17) $$\begin{array}{c} \langle \log^{2}x(t)\rangle -{\langle \log x(t)\rangle }^{2}=\sigma^{2}t. \end{array}$$

From Equations (A7), (14), and (16), we find the third and fourth log-moments,

(18) $$\begin{array}{c} \langle \log^{3}x(t)\rangle =\langle \log^{3}x_{0}\rangle +3\left\{\bar{\mu }\langle \log^{2}x_{0}\rangle +\sigma^{2}\langle \log x_{0}\rangle \right\}t+3\bar{\mu }\left\{\bar{\mu }\langle \log x_{0}\rangle +\sigma^{2}\right\}t^{2}+{\bar{\mu }}^{3}t^{3}, \end{array}$$

(19) $$\begin{array}{cc} \langle \log^{4}x(t)\rangle & =\langle \log^{4}x_{0}\rangle +2\left\{2\bar{\mu }\langle \log^{3}x_{0}\rangle +3\sigma^{2}\langle \log^{2}x_{0}\rangle \right\}t\\ & +3\left\{2\bar{\mu }\left(\bar{\mu }\langle \log^{2}x_{0}\rangle +2\sigma^{2}\langle \log x_{0}\rangle \right)+\sigma^{4}\right\}t^{2}+4{\bar{\mu }}^{2}\left\{\bar{\mu }\langle \log x_{0}\rangle +3\sigma^{2}/2\right\}t^{3}+{\bar{\mu }}^{4}t^{4}, \end{array}$$

respectively. The logarithm of the process in GBM has both skewness and excess kurtosis of 0, which can be shown by using y→logx in Equations (10) and (11), and the previous results for the first four log-moments. This implies that there is no asymmetry and excess “tailedness” in GBM. However, real world return distributions are known to exhibit both positive asymmetry (g>0) and fat-tailedness, i.e., positive excess kurtosis (κ>0).

### 2.2. Black–Scholes Formula

As previously said, perhaps the best-known application of GBM is in finance and, in particular, the BS model for pricing of European options. Formally, a European option is a contract that gives the buyer (the owner or holder of the option) the right, but not the obligation, to buy, or sell an underlying asset or instrument x(T) at a specified strike price *K* on a specified date *T*. The seller has the corresponding obligation to fulfil the transaction—to sell or buy—if the buyer (owner) “exercises” the option. An option that conveys to the owner the right to buy at a specific price is referred to as a call; an option that conveys the right of the owner to sell at a specific price is referred to as a put. Here, we are going to consider the valuation of call options, denoted as C(x,t), with the note that the derived results easily extend to put options.

In the modelling of financial assets, a standard assumption is that there is a risk-neutral distribution f(x,t) for the price of the asset. This measure is simply a probability distribution that takes into account the risk of future price fluctuations. Once a risk-neutral distribution is assigned, the value of the option is obtained by discounting the expectation of its value at the maturity *T* with respect to that distribution [6,42], i.e.,

(20) $$\begin{array}{c} C\left(x,t\right)=e^{-r(T-t)}\int_{K}^{\infty }\left(x\left(T\right)-K\right)f\left(x,T,|x_{0},0\right)dx, \end{array}$$

where *r* is the risk-free rate of return and $x_{0}$ is the asset price at the beginning (t=0). Notice that the integral is only calculated for the region of prices where the option has positive value, since, for asset price less than *K*, the option would not be exercised (i.e., its value is 0).

Assuming that the asset price follows GBM dynamics, Equations (20) and (4) can be combined in order to derive an analytical formula for the value of the call option in the BS model. In particular, the European option $C_{\mathrm{BS}}\left(x,t\right)$ (20) is a solution of the Black–Scholes equation, see, for example, [43],

(21) $$\begin{array}{c} \left(\frac{\partial }{\partial t}+\frac{\sigma^{2}x^{2}}{2}\frac{\partial^{2}}{\partial x^{2}}-r+r\;x\frac{\partial }{\partial x}\right)C_{\mathrm{BS}}\left(x,t\right)=0, \end{array}$$

with initial condition $C_{\mathrm{BS}}\left(x,T\right)=\max\left\{x-K,0\right\}$, x≥0, and boundary conditions $C_{\mathrm{BS}}\left(x=0,t\right)=0$, t≥T, and $C_{\mathrm{BS}}\left(x\to \infty ,t\right)\to x$. By using t=0 and T→t, one finds the equation

(22) $$\begin{array}{c} \frac{\partial }{\partial t}C_{\mathrm{BS}}\left(x,t\right)=\left(\frac{\sigma^{2}x^{2}}{2}\frac{\partial^{2}}{\partial x^{2}}-r+r\;x\frac{\partial }{\partial x}\right)C_{\mathrm{BS}}\left(x,t\right). \end{array}$$

with initial condition $C_{\mathrm{BS}}\left(x,t=0\right)=\max\left\{x-K,0\right\}$, x≥0, and boundary conditions $C_{\mathrm{BS}}\left(x=0,t\right)=0$, t≥0, and C(x→∞,t)→x.

The solution is

(23) $$\begin{array}{cc} C_{\mathrm{BS}}\left(x_{0},T,K,t\right) & =N\left(d_{1}\right)x\left(t\right)-N\left(d_{2}\right)Ke^{-\left(\mu -\frac{\sigma^{2}}{2}\right)\left(T-t\right)} \end{array}$$

(24) $$\begin{array}{cc} d_{1} & =\frac{1}{\sigma \sqrt{T-t}}\left[\log\frac{x(t)}{K}+\mu \left(T-t\right)\right] \end{array}$$

(25) $$\begin{array}{cc} d_{2} & =d_{1}-\sigma \sqrt{T-t}, \end{array}$$

where $N\left(x\right)=\frac{1}{\sqrt{2\pi }}\int_{-\infty }^{x}e^{-u^{2}/2}\;du$ is the cumulative distribution function of the Gaussian distribution with zero mean and unit variance. Put simply, the two terms in the BS formula describe the current price of the asset weighted by the probability that the investor will exercise its option at time *t* and the discounted price of the strike price weighted by its exercise probability. The terms $d_{1,2}$ can be seen as measures of the moneyness of the option and $N(d_{1,2})$ as probabilities that the option will expire, while its value is in the money. The neat BS formulation has allowed for the model to be widely applied in both theoretical investigations and empirical implementations. However, the BS model has failed to adequately reproduce a plethora of real world properties. In particular, theoretically predicted option prices with fixed values for drift μ and volatility σ via the BS model are known to significantly deviate from their respective market values in a plethora of cases. In order to deal with this problem, extensions of the BS model have emerged, which include a combination of the GBM with jumps [8,44] or with stochastic volatility [15,16].

### 2.3. Subdiffusive GBM

One of the reasons why the standard GBM is not able to explain empirical data is because it fails to reproduce periods of constant prices that appear on markets with low number of transactions. The price in these constant periods can be described as a trapped particle, which, in physical systems, manifests anomalous diffusion (subdiffusion) [35,36]. To deal with this problem, the so-called subdiffusive GBM (sGBM) has been developed by Magdziarz [28], by using the subordination approach. The corresponding equation for the sGBM becomes the following fractional Fokker–Planck equation [28] (see also [29])

(26) $$\begin{array}{cc} \frac{\partial }{\partial t}f_{\alpha }\left(x,t\right)={}_{\mathrm{RL}}D_{t}^{1-\alpha } & \left[-\mu \frac{\partial }{\partial x}xf_{\alpha }\left(x,t\right)+\frac{\sigma^{2}}{2}\frac{\partial^{2}}{\partial x^{2}}x^{2}f_{\alpha }\left(x,t\right)\right], \end{array}$$

where

(27) $$\begin{array}{c} {}_{\mathrm{RL}}D_{t}^{\nu }f\left(t\right)=\frac{1}{\Gamma (1-\nu )}\frac{d}{dt}\int_{0}^{t}{\left(t-t^{\prime }\right)}^{-\nu }f\left(t^{\prime }\right)\;dt^{\prime } \end{array}$$

is the Riemann–Liouville fractional derivative of order 0<ν<1 [45]. The Laplace transform of the Riemann–Liouville fractional derivative of a given function reads $L\left\{{}_{\mathrm{RL}}D_{t}^{\nu }f\left(t\right)\right\}\left(s\right)=s^{\nu }L\left\{f(t)\right\}\left(s\right)-{}_{\mathrm{RL}}I_{t}^{1-\nu }f\left(0+\right)$, where ${}_{\mathrm{RL}}I_{t}^{\nu }f\left(t\right)=\frac{1}{\Gamma (\nu )}\int_{0}^{t}{\left(t-t^{\prime }\right)}^{\nu -1}f\left(t^{\prime }\right)\;dt^{\prime }$ is the Riemann–Liouville fractional integral. In order to avoid the somewhat unusual fractional initial condition, alternatively, we could alternatively use the integral version of the equation [46]

(28) $$\begin{array}{cc} f_{\alpha }\left(x,t\right)-f_{\alpha }\left(x,0\right)={}_{\mathrm{RL}}D_{t}^{-\alpha } & \left[-\mu \frac{\partial }{\partial x}xf_{\alpha }\left(x,t\right)+\frac{\sigma^{2}}{2}\frac{\partial^{2}}{\partial x^{2}}x^{2}f_{\alpha }\left(x,t\right)\right], \end{array}$$

where $L\left\{{}_{\mathrm{RL}}D_{t}^{-\alpha }f\left(t\right)\right\}\left(s\right)=s^{-\alpha }L\left\{f\left(t\right)\right\}\left(s\right)$. In Ref. [29], the time fractional Fokker–Planck Equation (26) for sGBM is derived within the CTRW theory for a particle on a geometric lattice in the presence of a logarithmic potential.

Here we note that the fractional Fokker–Planck Equation (26) can be obtained using the Langevin equation approach [47], i.e., by considering a CTRW model that was described by a coupled Langevin equations [48],

(29) $$\begin{array}{cc} \; & \frac{d}{du}x\left(u\right)=\mu \;x\left(u\right)+\sigma \;x\left(u\right)\;\xi \left(u\right), \end{array}$$

(30) $$\begin{array}{cc} \; & \frac{d}{du}T\left(u\right)=\zeta \left(u\right). \end{array}$$

Therefore, x(t) is parametrised in terms of the number of steps *u*, and the connection to the physical time *t* is given by $T\left(u\right)=\int_{0}^{u}\tau \left(u^{\prime }\right)\;du$, where τ(u) is a total of individual waiting times τ for each step. In mathematical terms, this is called subordination [49,50,51]. The noise ξ(u) is a white noise with zero mean and correlation $\langle \xi \left(u\right)\xi \left(u^{\prime }\right)\rangle =2\delta \left(u-u^{\prime }\right)$, while ζ(u) is one-sided α-stable Lévy noise with the stable index 0<α<1. The inverse process S(t) of the one-sided α-stable Levy process T(u) with a characteristic function $\langle e^{-sT(u)}\rangle =e^{-s^{\alpha }u}$ is given by $S\left(t\right)=\inf\left\{u>0:T(u)>t\right\}$, i.e., it represents a collection of first passage times [47]. The CTRW is defined by the subordinated process X(t)=x(S(t)).

The PDF h(u,t) of the inverse process S(t) can be found from the relation [47]

(31) $$\begin{array}{c} h\left(u,t\right)=-\frac{\partial }{\partial u}\Theta \left(t-T(u)\right), \end{array}$$

where Θ(z) is the Heaviside theta function. The Laplace transform then yields

(32) $$\begin{array}{c} \hat{h}\left(u,s\right)=-\frac{\partial }{\partial u}\frac{1}{s}\;\langle \int_{0}^{\infty }\delta \left(t-T(u)\right)e^{-st}\;dt\rangle =-\frac{\partial }{\partial u}\frac{1}{s}\langle e^{-sT(u)}\rangle =-\frac{\partial }{\partial u}\frac{1}{s}e^{-s^{\alpha }u}=s^{\alpha -1}e^{-s^{\alpha }u}. \end{array}$$

Hence, $f_{\alpha }\left(x,t\right)=\langle \delta \left(x-X\left(t\right)\right)\rangle =\langle \delta (x-X\left(S\left(t\right)\right)\rangle =\int_{0}^{\infty }f\left(x,u\right)\;h\left(u,t\right)\;dt$, from where one can easily arrive to the fractional Fokker–Planck Equation (26).

The mean value for sGBM is given by [29,48]

(33) $$\begin{array}{c} \langle x\left(t\right)\rangle =x_{0}\;E_{\alpha }\left(\mu t^{\alpha }\right), \end{array}$$

where $E_{\alpha }\left(z\right)$ is the one parameter Mittag–Leffler (ML) function [35,45]

(34) $$\begin{array}{c} E_{\alpha }\left(z\right)=\sum_{k=0}^{\infty }\frac{z^{k}}{\Gamma (\alpha k+1)}, \end{array}$$

with (z∈C;R(α)>0), and Γ(·) is the Gamma function. The ML function is a generalisation of the exponential function, since $E_{1}\left(z\right)=e^{z}$. The Laplace transform of the one parameter ML function reads $L\left\{E_{\alpha }\left(at^{\alpha }\right)\right\}\left(s\right)=\frac{s^{\alpha -1}}{s^{\alpha }-a}$. The asymptotic behaviour of the mean is given by

(35) $$\begin{array}{c} \langle x(t)\rangle ∼x_{0}\;\left\{\begin{array}{ccc} & 1+\mu t^{\alpha }/\Gamma \left(1+\alpha \right)∼e^{\mu t^{\alpha }/\Gamma \left(1+\alpha \right)},\; & t\ll 1,\\ \; & \alpha^{-1}e^{\mu^{1/\alpha }t},\; & t\gg 1. \end{array}\right. \end{array}$$

For the short time limit, we use the first two terms from the series expansion of the ML function (34), while, for the long time limit, we apply its asymptotic expansion formula $E_{\alpha }\left(z\right)∼\frac{1}{\alpha }e^{z^{1/\alpha }}$, z≫1 [45,52]. Here, we note that the asymptotic behaviour of the ML function with negative argument has a power-law form, i.e., $E_{\alpha }\left(-z^{\alpha }\right)∼\frac{z^{-\alpha }}{\Gamma (1-\alpha )}$ for z≪1 and 0<α<2 [45,52].

The MSD also is given through the one parameter ML function [29,48]

(36) $$\begin{array}{cc} \langle x^{2}\left(t\right)\rangle \; & =x_{0}^{2}\;E_{\alpha }\left(\left(\sigma^{2}+2\mu \right)t^{\alpha }\right)\\ & ∼\langle x^{2}\left(0\right)\rangle \left\{\begin{array}{ccc} & 1+\left(\sigma^{2}+2\mu \right)t^{\alpha }/\Gamma \left(1+\alpha \right)∼e^{\left(\sigma^{2}+2\mu \right)t^{\alpha }/\Gamma \left(1+\alpha \right)},\; & t\ll 1,\\ \; & \alpha^{-1}e^{{\left(\sigma^{2}+2\mu \right)}^{1/\alpha }t},\; & t\gg 1. \end{array}\right. \end{array}$$

From here, one concludes that sGBM is an exponentially fast process. Moreover, the third and fourth moments, respectively, become

(37) $$\begin{array}{c} \langle x^{3}\left(t\right)\rangle =x_{0}^{3}\;E_{\alpha }\left(\left(3\sigma^{2}+3\mu \right)t^{\alpha }\right), \end{array}$$

(38) $$\begin{array}{c} \langle x^{4}\left(t\right)\rangle =x_{0}^{4}\;E_{\alpha }\left(\left(6\sigma^{2}+4\mu \right)t^{\alpha }\right). \end{array}$$

The first log-moment has the form [29]

(39) $$\begin{array}{c} \langle \log x(t)\rangle =\langle \log x_{0}\rangle +\bar{\mu }\;\int_{0}^{t}\frac{t^{\prime \alpha -1}}{\Gamma (\alpha )}\;dt^{\prime }=\langle \log x_{0}\rangle +\bar{\mu }\;\frac{t^{\alpha }}{\Gamma (1+\alpha )}, \end{array}$$

which gives a power-law dependence with respect to time of the expectation of the log return with period Δt, i.e., [29]

(40) $$\begin{array}{c} \frac{1}{\Delta t}\langle \log\left(x(t+\Delta t)/x(t)\right)\rangle \underset{\Delta t\to 0}{∼}\bar{\mu }\;\frac{t^{\alpha -1}}{\Gamma (\alpha )}. \end{array}$$

Such models have been used, for example, to explain the dynamics of an asset before a market crash [53]. The second log-moment becomes [29]

(41) $$\begin{array}{c} \langle \log^{2}x(t)\rangle =\langle \log^{2}x_{0}\rangle +\left[2\bar{\mu }\langle \log x_{0}\rangle +\sigma^{2}\right]\frac{t^{\alpha }}{\Gamma (1+\alpha )}+2{\bar{\mu }}^{2}\frac{t^{2\alpha }}{\Gamma (1+2\alpha )}. \end{array}$$

from where, for the log-variance, one finds [29]

(42) $$\begin{array}{c} \langle \log^{2}x(t)\rangle -{\langle \log x(t)\rangle }^{2}=\sigma^{2}\frac{t^{\alpha }}{\Gamma (1+\alpha )}+{\bar{\mu }}^{2}\left[\frac{2}{\Gamma (1+2\alpha )}-\frac{1}{\Gamma^{2}\left(1+\alpha \right)}\right]t^{2\alpha }, \end{array}$$

which, in the long time, scales as $t^{2\alpha }$ (0<α<1), contrary to the linear scaling *t* for regular GBM (α=1). For the third and fourth log-moments, we obtain

(43) $$\begin{array}{cc} \langle \log^{3}x(t)\rangle & =\langle \log^{3}x_{0}\rangle +3\left\{\bar{\mu }\langle \log^{2}x_{0}\rangle +\sigma^{2}\langle \log x_{0}\rangle \right\}\frac{t^{\alpha }}{\Gamma (\alpha +1)}\\ & +6\bar{\mu }\left\{\bar{\mu }\langle \log x_{0}\rangle +\sigma^{2}\right\}\frac{t^{2\alpha }}{\Gamma (2\alpha +1)}+6{\bar{\mu }}^{3}\frac{t^{3\alpha }}{\Gamma (3\alpha +1)}, \end{array}$$

(44) $$\begin{array}{cc} \langle \log^{4}x(t)\rangle \; & =\langle \log^{4}x_{0}\rangle +2\left\{2\bar{\mu }\langle \log^{3}x_{0}\rangle +3\sigma^{2}\langle \log^{2}x_{0}\rangle \right\}\frac{t^{\alpha }}{\Gamma (\alpha +1)}\\ & +6\left\{2\bar{\mu }\left(\bar{\mu }\langle \log^{2}x_{0}\rangle +2\sigma^{2}\langle \log x_{0}\rangle \right)+\sigma^{4}\right\}\frac{t^{2\alpha }}{\Gamma (2\alpha +1)}\\ & +24{\bar{\mu }}^{2}\left\{\bar{\mu }\langle \log x_{0}\rangle +3\sigma^{2}/2\right\}\frac{t^{3\alpha }}{\Gamma (3\alpha +1)}+24{\bar{\mu }}^{4}\frac{t^{4\alpha }}{\Gamma (4\alpha +1)}. \end{array}$$

## 3. Generalised GBM

In this section, we consider a generalisation of GBM, under which the standard and subdiffusive GBM arise as special cases, by using the subordination approach. Here, we present analytical expressions for the first four moments and log-moments of the process for a variety of special cases. They are thoroughly analysed in the numerical experiments section.

The continuous time random walk approach to the corresponding Fokker–Planck equation is given in Appendix B in detail. The same Fokker–Planck equation can be obtained using the coupled Langevin equations approach [47], as given in Equations (29) and (30), where the waiting times are given by $\langle e^{-sT(u)}\rangle =e^{-u\hat{\Psi }\left(s\right)}$, with $\hat{\Psi }\left(s\right)=1/\hat{\eta }\left(s\right)$.

### 3.1. Subordination Approach

The generalisation of GBM that we consider is the model introduced by Magdziarz and Gajda [37] in the form of a stochastic process

(45) $$\begin{array}{c} X\left(t\right)=x\left(S(t)\right), \end{array}$$

where X(t) is the *generalised* GBM (gGBM), $S\left(t\right)=\inf\left\{u>0:T(u)>t\right\}$ is the operational time, and T(u) is an infinite divisible process, i.e., a strictly increasing Lévy motion with [37]

$$\langle e^{-sT(u)}\rangle =e^{-u\hat{\Psi }\left(s\right)},$$

and $\hat{\Psi }\left(s\right)$ is the Lévy exponent [28,37,39]. Here we consider $\hat{\Psi }\left(s\right)=1/\hat{\eta }\left(s\right)$. The current process should not be confused with the generalised grey Brownian motion, see Ref. [54,55,56].

Next we find the PDF of gGBM which subordinates the processes from the time scale *t* (physical time) to the GBM on a time scale *u* (operational time). Specifically, the PDF P(x,t) of a given random process X(t) can be represented as [28,46,57,58,59]

(46) $$\begin{array}{c} P\left(x,t\right)=\int_{0}^{\infty }f\left(x,u\right)h\left(u,t\right)\;du, \end{array}$$

where f(x,u) satisfies the Fokker–Planck Equation (3) for the standard GBM. The function h(u,t) is the PDF subordinating the random process X(t) to the standard GBM. In Laplace space, Equation (46) reads

(47) $$\begin{array}{cc} \hat{P}\left(x,s\right)=L\left\{P(x,t)\right\} & =\int_{0}^{\infty }e^{-st}P\left(x,t\right)\;dt=\int_{0}^{\infty }f\left(x,u\right)\hat{h}\left(u,s\right)\;du, \end{array}$$

where $\hat{h}\left(u,s\right)=L\left\{h(u,t)\right\}$. By considering

(48) $$\begin{array}{c} \hat{h}\left(u,s\right)=\frac{\hat{\Psi }\left(s\right)}{s}e^{-u\hat{\Psi }\left(s\right)}=\frac{1}{s\hat{\eta }\left(s\right)}e^{-\frac{u}{\hat{\eta }\left(s\right)}}, \end{array}$$

we then have

(49) $$\begin{array}{c} \hat{P}\left(x,s\right)=\frac{1}{s\hat{\eta }\left(s\right)}\int_{0}^{\infty }f\left(x,u\right)e^{-\frac{u}{\hat{\eta }\left(s\right)}}\;du=\frac{1}{s\hat{\eta }\left(s\right)}\;\hat{f}\left(x,\frac{1}{\hat{\eta }\left(s\right)}\right). \end{array}$$

By Laplace transform of the Fokker–Planck Equation (3) for the GBM, and using relation (49), one finds that the PDF P(x,s) satisfies

(50) $$\begin{array}{cc} s\hat{P}\left(x,s\right)-P\left(x,0\right)=s\;\hat{\eta }\left(s\right) & \left[-\mu \frac{\partial }{\partial x}x\hat{P}\left(x,s\right)+\frac{\sigma^{2}}{2}\frac{\partial^{2}}{\partial x^{2}}x^{2}\hat{P}\left(x,s\right)\right]. \end{array}$$

After applying an inverse Laplace transform, we arrive at the generalised Fokker–Planck equation (see Refs. [37,48], where one-sided α-stable waiting times are considered in detail)

(51) $$\begin{array}{cc} \frac{\partial }{\partial t}P\left(x,t\right)=\frac{\partial }{\partial t}\int_{0}^{t}\eta \left(t-t^{\prime }\right) & \left[-\mu \frac{\partial }{\partial x}xP\left(x,t^{\prime }\right)+\frac{\sigma^{2}}{2}\frac{\partial^{2}}{\partial x^{2}}x^{2}P\left(x,t^{\prime }\right)\right]dt^{\prime }, \end{array}$$

where η(t) is a so-called memory kernel. One observes that, for η(t)=1, we arrive at the Fokker–Planck Equation (3) for the GBM, and for $\eta \left(t\right)=\frac{t^{\alpha -1}}{\Gamma (\alpha )}$ at the time fractional Fokker–Planck Equation (26) for the sGBM. From Equations (47) and (48), we find for the PDF in the Laplace domain, see also Ref. [48],

(52) $$\begin{array}{cc} \hat{P}\left(x,s\right) & =\int_{0}^{\infty }\frac{1}{x\sqrt{2\pi \sigma^{2}u}}\times \exp\left(-\frac{{\left[\log x-\log x_{0}-\bar{\mu }u\right]}^{2}}{2\sigma^{2}u}\right)\frac{1}{s\hat{\eta }\left(s\right)}e^{-\frac{u}{\hat{\eta }\left(s\right)}}\;du\\ & =\frac{1/[s\hat{\eta }\left(s\right)]}{x\sqrt{{\bar{\mu }}^{2}+2\sigma^{2}/\hat{\eta }\left(s\right)}}\left\{\begin{array}{ccc} & \exp\left(-\frac{\log x-\log x_{0}}{\sigma^{2}}\left[\sqrt{{\bar{\mu }}^{2}+2\sigma^{2}/\hat{\eta }\left(s\right)}-\bar{\mu }\right]\right),\; & x>x_{0},\\ \; & 1,\; & x=x_{0},\\ \; & \exp\left(\frac{\log x-\log x_{0}}{\sigma^{2}}\left[\sqrt{{\bar{\mu }}^{2}+2\sigma^{2}/\hat{\eta }\left(s\right)}+\bar{\mu }\right]\right),\; & x<x_{0}, \end{array}\right. \end{array}$$

**Remark** **1.** *Here, we note that there are restrictions on the choice of the memory kernel η(t) since the PDF (46) should be non-negative. From the subordination integral it follows that the subordination function h(u,t) should be non-negative, which, according to the Bernstein theorem, means that its Laplace transform (48) should be a completely monotone function [60]. Therefore, the PDF (46) will be non-negative if $1/[s\hat{\eta }\left(s\right)]$ is a completely monotone function, and $1/\hat{\eta }\left(s\right)$ is a Bernstein function, see Refs. [61,62].*

**Remark** **2.** *We note that Equation (50) can be written in an equivalent form as*

(53) $$\begin{array}{cc} \int_{0}^{t}\gamma \left(t-t^{\prime }\right)\frac{\partial }{\partial t^{\prime }}P\left(x,t^{\prime }\right)\;dt^{\prime } & =-\mu \frac{\partial }{\partial x}xP\left(x,t\right)+\frac{\sigma^{2}}{2}\frac{\partial^{2}}{\partial x^{2}}x^{2}P\left(x,t\right), \end{array}$$

*where the memory kernel γ(t) is connected to η(t) in Laplace space as γ(s)=1/[sη(s)], see Ref. [61]. From this relation, we find that for GBM (η(t)=1, i.e., $\hat{\eta }\left(s\right)=1/s$) the memory kernel γ(t) is given by $\gamma \left(t\right)=L^{-1}\left\{s^{-1}{\hat{\eta }}^{-1}\left(s\right)\right\}=L^{-1}\left\{1\right\}=\delta \left(t\right)$. For sGBM ($\eta \left(t\right)=t^{\alpha -1}/\Gamma \left(\alpha \right)$, i.e., $\hat{\eta }\left(s\right)=s^{-\alpha }$) the memory kernel becomes $\gamma \left(t\right)=L^{-1}\left\{s^{\alpha -1}\right\}=t^{-\alpha }/\Gamma \left(1-\alpha \right)$, and, thus, Equation (53) reads*

(54) $$\begin{array}{cc} {}_{C}D_{t}^{\alpha }P\left(x,t\right) & =-\mu \frac{\partial }{\partial x}xP\left(x,t\right)+\frac{\sigma^{2}}{2}\frac{\partial^{2}}{\partial x^{2}}x^{2}P\left(x,t\right), \end{array}$$

*where*

(55) $$\begin{array}{c} {}_{C}D_{t}^{\nu }f\left(t\right)=\frac{1}{\Gamma (1-\nu )}\int_{0}^{t}{\left(t-t^{\prime }\right)}^{-\nu }\frac{d}{dt^{\prime }}f\left(t^{\prime }\right)\;dt^{\prime } \end{array}$$

*is the Caputo fractional derivative of order 0<ν<1 [45]. The Laplace transform of the Caputo derivative of a given function reads $L\left\{{}_{C}D_{t}^{\nu }f\left(t\right)\right\}\left(s\right)=s^{\nu }L\left\{f(t)\right\}\left(s\right)-s^{\nu -1}f\left(0+\right)$. We note that, with the appropriate restrictions for η(t) and γ(t), both formulations are equivalent.*

**Remark** **3.** *For $\eta \left(t\right)=\frac{t^{\alpha -1}}{\Gamma (\alpha )}$, 0<α<1, gGBM corresponds to sGBM. Using the subordination approach one finds [28]*

(56) $$\begin{array}{c} \hat{h}\left(u,s\right)=s^{\alpha -1}e^{-us^{\alpha }}=s^{\alpha -1}H_{0,1}^{1,0}\left[u\;s^{\alpha }\left|\begin{array}{c} -\\ \left(0,1\right) \end{array}\right.\right], \end{array}$$

*where $H_{p,q}^{m,n}\left(z\right)$ is the Fox H-function, see Appendix D. By inverse Laplace transform (A42), one obtains [28]*

(57) $$\begin{array}{c} h\left(u,t\right)=L^{-1}\left\{\hat{h}\left(u,s\right)\right\}=t^{-\alpha }\;H_{1,1}^{1,0}\left[\frac{u}{t^{\alpha }}\left|\begin{array}{c} \left(1-\alpha ,\alpha \right)\\ \left(0,1\right) \end{array}\right.\right]=\frac{1}{u}\;H_{1,1}^{1,0}\left[\frac{u}{t^{\alpha }}\left|\begin{array}{c} \left(1,\alpha \right)\\ \left(1,1\right) \end{array}\right.\right], \end{array}$$

*where we applied property (A43). The solution in Laplace space then becomes*

(58) $$\begin{array}{cc} \hat{P}\left(x,s\right) & =\int_{0}^{\infty }\frac{1}{x\sqrt{2\pi \sigma^{2}u}}\times \exp\left(-\frac{{\left[\log x-\log x_{0}-\bar{\mu }u\right]}^{2}}{2\sigma^{2}u}\right)s^{\alpha -1}e^{-us^{\alpha }}\;du\\ & =\frac{s^{\alpha -1}}{x\sqrt{{\bar{\mu }}^{2}+2\sigma^{2}s^{\alpha }}}\times \left\{\begin{array}{ccc} & \exp\left(-\frac{\log x-\log x_{0}}{\sigma^{2}}\left[\sqrt{{\bar{\mu }}^{2}+2\sigma^{2}s^{\alpha }}-\bar{\mu }\right]\right),\; & x>x_{0},\\ & 1,\; & x=x_{0},\\ \; & \exp\left(\frac{\log x-\log x_{0}}{\sigma^{2}}\left[\sqrt{{\bar{\mu }}^{2}+2\sigma^{2}s^{\alpha }}+\bar{\mu }\right]\right),\; & x<x_{0}, \end{array}\right. \end{array}$$

*which is obtained in Ref. [48] in a similar way. From here, we can plot the PDF using numerical inverse Laplace transform techniques.*

### 3.2. Generalised BS Formula

If we consider that the asset price follows a gGBM, then the generalised BS (gBS) formula for the option price is [37]

(59) $$\begin{array}{c} C_{\mathrm{gBS}}\left(x,t\right)={\langle e^{-r(S(T)-t)}\left(x\left(S\left(T\right)\right)-K\right)\rangle }_{x}=\int_{0}^{\infty }C_{\mathrm{BS}}\left(x,u\right)\;h\left(u,T\right)\;du, \end{array}$$

where $C_{\mathrm{BS}}\left(x,t\right)$ is taken from the BS Formula (25), and h(x,T) is the subordination function defined by Equation (48) in the Laplace domain. By Laplace transform one finds

(60) $$\begin{array}{c} {\hat{C}}_{\mathrm{gBS}}\left(x,s\right)=\frac{1}{s\hat{\eta }\left(s\right)}\;{\hat{C}}_{\mathrm{BS}}\left(x,1/\hat{\eta }\left(s\right)\right). \end{array}$$

Therefore, from Equation (22), the corresponding equation for the option price becomes [48]

(61) $$\begin{array}{c} \frac{\partial }{\partial t}C_{\mathrm{gBS}}\left(x,t\right)=\frac{\partial }{\partial t}\int_{0}^{t}\eta \left(t-t^{\prime }\right)\left(\frac{\sigma^{2}x^{2}}{2}\frac{\partial^{2}}{\partial x^{2}}-r+r\;x\frac{\partial }{\partial x}\right)C_{\mathrm{gBS}}\left(x,t^{\prime }\right)\;dt. \end{array}$$

### 3.3. Calculation of Moments

The *n*th moment $\langle X^{n}\left(t\right)\rangle =\int_{0}^{\infty }x^{n}\;P\left(x,t\right)\;dx$ can be calculated by multiplying both sides of Equation (51) by $x^{n}$ and integration over *x*, see Appendix C. In the Laplace domain, this results in

(62) $$\begin{array}{c} \langle {\hat{X}}^{n}\left(s\right)\rangle =x_{0}^{n}\;\frac{s^{-1}}{1-\hat{\eta }\left(s\right)\left[\frac{\sigma^{2}}{2}n\left(n-1\right)+\mu \;n\right]}. \end{array}$$

From this result, we reproduce the normalisation condition $\langle x^{0}\left(t\right)\rangle =\langle x_{0}^{0}\rangle =1$. The general results for the first four moments in terms of the memory kernel become

(63) $$\begin{array}{c} \langle \hat{X}\left(s\right)\rangle =x_{0}\;\frac{s^{-1}}{1-\mu \hat{\eta }\left(s\right)}, \end{array}$$

(64) $$\begin{array}{c} \langle {\hat{X}}^{2}\left(s\right)\rangle =x_{0}^{2}\;\frac{s^{-1}}{1-\left(\sigma^{2}+2\mu \right)\hat{\eta }\left(s\right)}, \end{array}$$

(65) $$\begin{array}{c} \langle {\hat{X}}^{3}\left(s\right)\rangle =x_{0}^{3}\;\frac{s^{-1}}{1-3\hat{\eta }\left(s\right)\left[\sigma^{2}+\mu \right]}, \end{array}$$

(66) $$\begin{array}{c} \langle {\hat{X}}^{4}\left(s\right)\rangle =x_{0}^{4}\;\frac{s^{-1}}{1-4\hat{\eta }\left(s\right)\left[\frac{3\sigma^{2}}{2}+\mu \right]}. \end{array}$$

The log-moments $\langle \log^{n}x(t)\rangle =\int_{0}^{\infty }\log^{n}x\;P\left(x,t\right)\;dx$, can also be calculated exactly through the memory kernel, see Appendix C. The normalisation condition is satisfied, i.e., $\langle \log^{0}x(t)\rangle =1$, while the log-mean reads

(67) $$\begin{array}{c} \langle \log x(t)\rangle =\langle \log x_{0}\rangle +\bar{\mu }\int_{0}^{t}\eta \left(t^{\prime }\right)\;dt^{\prime }. \end{array}$$

From here, we find, for the expectation of the periodic log return with period Δt

(68) $$\begin{array}{cc} \frac{1}{\Delta t}\langle \log\left(x(t+\Delta t)/x(t)\right)\rangle & =\bar{\mu }\frac{1}{\Delta t}\int_{t}^{t+\Delta t}\eta \left(t^{\prime }\right)\;dt^{\prime }=\bar{\mu }\frac{I(t+\Delta t)-I(t)}{\Delta t}\underset{\Delta t\to 0}{∼}\bar{\mu }\;\eta \left(t\right), \end{array}$$

where I(t)=∫η(t)dt, i.e., $I^{\prime }\left(t\right)=\eta \left(t\right)$. Therefore, the expectation of the periodic log returns behaves as the rate of the first log-moment,

(69) $$\begin{array}{c} \frac{1}{\Delta t}\langle \log\left(x(t+\Delta t)/x(t)\right)\rangle \underset{\Delta t\to 0}{∼}\frac{d}{dt}\langle \log x(t)\rangle , \end{array}$$

which is proportional to the memory kernel η(t). Moreover, for the second log-moment, we find

(70) $$\begin{array}{cc} \langle \log^{2}x(t)\rangle & =\langle \log^{2}x_{0}\rangle +\int_{0}^{t}\eta \left(t-t^{\prime }\right)\left\{2\bar{\mu }\left[\langle \log x_{0}\rangle +\bar{\mu }\int_{0}^{t^{\prime }}\eta \left(t^{\prime \prime }\right)\;dt^{\prime \prime }\right]+\sigma^{2}\right\}dt^{\prime }, \end{array}$$

from where the log-variance becomes

(71) $$\begin{array}{cc} \langle \log^{2}x(t)\rangle -{\langle \log x(t)\rangle }^{2} & =\sigma^{2}\int_{0}^{t}\eta \left(t^{\prime }\right)\;dt^{\prime }\\ & +{\bar{\mu }}^{2}\left[2\int_{0}^{t}\eta \left(t-t^{\prime }\right)\left(\int_{0}^{t^{\prime }}\eta \left(t^{\prime \prime }\right)\;dt^{\prime \prime }\right)dt^{\prime }-{\left(\int_{0}^{t}\eta \left(t^{\prime }\right)\;dt^{\prime }\right)}^{2}\right]. \end{array}$$

The general results for third and fourth moments are given in Appendix C.

From all of these general formulas, one can easily recover the previous results for the standard GBM (η(t)=1, i.e., $\hat{\eta }\left(s\right)=1/s$) and sGBM ($\eta \left(t\right)=t^{\alpha -1}/\Gamma \left(\alpha \right)$, i.e., $\hat{\eta }\left(s\right)=s^{-\alpha }$, 0<α<1).

### 3.4. Exponentially Truncated Subdiffusive GBM

As an example for another memory kernel in gGBM, we consider a power-law memory kernel with exponential truncation,

(72) $$\begin{array}{c} \eta \left(t\right)=\frac{t^{\alpha -1}}{\Gamma (\alpha )}e^{-\frac{t}{\tau }}, \end{array}$$

where τ is a characteristic crossover time scale, 0<α<1. Such forms are important in many real-world applications, in which the scale-free nature of the waiting time dynamics is broken at macroscopic times t≫τ [61]. Therefore,

(73) $$\begin{array}{c} \hat{\eta }\left(s\right)={\left(s+\tau^{-1}\right)}^{-\alpha }, \end{array}$$

where we use the shift rule of the Laplace transform, $L\left\{e^{-at}f\left(t\right)\right\}=\hat{F}\left(s+a\right)$, for $\hat{F}\left(s\right)=L\left\{f(t)\right\}$.

The mean value reads,

(74) $$\begin{array}{cc} \langle x(t)\rangle & =x_{0}\;L^{-1}\left\{\frac{s^{-1}}{1-\mu {\left(s+\tau^{-1}\right)}^{-\alpha }}\right\}\left(t\right)=x_{0}\;L^{-1}\left\{\frac{s+\tau^{-1}}{s}\frac{{\left(s+\tau^{-1}\right)}^{\alpha -1}}{{\left(s+\tau^{-1}\right)}^{\alpha }-\mu }\right\}\left(t\right)\\ & =x_{0}\left[e^{-t/\tau }E_{\alpha }\left(\mu t^{\alpha }\right)+\tau^{-1}\int_{0}^{t}e^{-t^{\prime }/\tau }E_{\alpha }\left(\mu t^{\prime \alpha }\right)dt^{\prime }\right], \end{array}$$

and the MSD is

(75) $$\begin{array}{cc} \langle x^{2}\left(t\right)\rangle & =x_{0}^{2}\;L^{-1}\left\{\frac{s^{-1}}{1-\left(\sigma^{2}+2\mu \right){\left(s+\tau^{-1}\right)}^{-\alpha }}\right\}\left(t\right)=x_{0}^{2}\;L^{-1}\left\{\frac{s+\tau^{-1}}{s}\frac{{\left(s+\tau^{-1}\right)}^{\alpha -1}}{{\left(s+\tau^{-1}\right)}^{\alpha }-\left(\sigma^{2}+2\mu \right)}\right\}\left(t\right)\\ & =x_{0}^{2}\left[e^{-t/\tau }E_{\alpha }\left(\left(\sigma^{2}+2\mu \right)t^{\alpha }\right)+\tau^{-1}\int_{0}^{t}e^{-t^{\prime }/\tau }E_{\alpha }\left(\left(\sigma^{2}+2\mu \right)t^{\prime \alpha }\right)dt^{\prime }\right]. \end{array}$$

The third and fourth moments become

(76) $$\begin{array}{cc} \langle x^{3}\left(t\right)\rangle & =x_{0}^{3}\;L^{-1}\left\{\frac{s^{-1}}{1-3{\left(s+\tau^{-1}\right)}^{-\alpha }\left(\sigma^{2}+\mu \right)}\right\}\left(t\right)=x_{0}^{3}\;L^{-1}\left\{\frac{s+\tau^{-1}}{s}\frac{{\left(s+\tau^{-1}\right)}^{\alpha -1}}{{\left(s+\tau^{-1}\right)}^{\alpha }-3\left(\sigma^{2}+\mu \right)}\right\}\left(t\right)\\ & =x_{0}^{3}\;\left[e^{-t/\tau }E_{\alpha }\left(\left(3\sigma^{2}+3\mu \right)t^{\alpha }\right)+\tau^{-1}\int_{0}^{t}e^{-t^{\prime }/\tau }E_{\alpha }\left(\left(3\sigma^{2}+3\mu \right)t^{\prime \alpha }\right)dt^{\prime }\right], \end{array}$$

(77) $$\begin{array}{cc} \langle x^{4}\left(t\right)\rangle & =x_{0}^{4}\;L^{-1}\left\{\frac{s^{-1}}{1-{\left(s+\tau^{-1}\right)}^{-\alpha }\left(6\sigma^{2}+4\mu \right)}\right\}\left(t\right)=x_{0}^{4}\;L^{-1}\left\{\frac{s+\tau^{-1}}{s}\frac{{\left(s+\tau^{-1}\right)}^{\alpha -1}}{{\left(s+\tau^{-1}\right)}^{\alpha }-\left(6\sigma^{2}+4\mu \right)}\right\}\left(t\right)\\ & =x_{0}^{4}\left[e^{-t/\tau }E_{\alpha }\left(\left(6\sigma^{2}+4\mu \right)t^{\alpha }\right)+\tau^{-1}\int_{0}^{t}e^{-t^{\prime }/\tau }E_{\alpha }\left(\left(6\sigma^{2}+4\mu \right)t^{\prime \alpha }\right)dt^{\prime }\right], \end{array}$$

respectively.

From the general result for the log-mean, we find that

(78) $$\begin{array}{c} \langle \log x(t)\rangle =\langle \log x_{0}\rangle +\bar{\mu }\;\tau^{\alpha }\frac{\gamma (\alpha ,t/\tau )}{\Gamma (\alpha )}=\langle \log x_{0}\rangle +\bar{\mu }\;e^{-t/\tau }\;t^{\alpha }E_{1,\alpha +1}\left(t/\tau \right), \end{array}$$

where (z,β∈C; R(α)>0). Here, $\gamma \left(a,z\right)=\int_{0}^{z}t^{a-1}e^{-t}\;dt=\Gamma \left(a\right)e^{-z}\;z^{a}E_{1,a+1}\left(z\right)$ is the incomplete gamma function, and

(79) $$\begin{array}{c} E_{\alpha ,\beta }\left(z\right)=\sum_{k=0}^{\infty }\frac{z^{k}}{\Gamma (\alpha k+\beta )}. \end{array}$$

is the two parameter ML function [45]. Note that, the Laplace transform of the two parameter ML function reads $L\left\{t^{\beta -1}E_{\alpha ,\beta }\left(at^{\alpha }\right)\right\}\left(s\right)=\frac{s^{\alpha -\beta }}{s^{\alpha }-a}$. The asymptotic expansion formula for the two parameter ML function is $E_{\alpha ,\beta }\left(z\right)∼\frac{1}{\alpha }\;e^{z^{1/\alpha }}z^{(1-\beta )/\alpha }$, z≫1 [45,52], while the asymptotic behaviour for negative arguments is given by power-law decay, $E_{\alpha ,\beta }\left(-z^{\alpha }\right)∼\frac{z^{-\alpha }}{\Gamma (\beta -\alpha )}$, z≫1 [45,52].

For the expectation of the periodic log return with period Δt, we find

(80) $$\begin{array}{c} \frac{1}{\Delta t}\langle \log\left(x(t+\Delta t)/x(t)\right)\rangle \underset{\Delta t\to 0}{∼}\bar{\mu }\;\frac{t^{\alpha -1}}{\Gamma (\alpha )}e^{-t/\tau }=\frac{d}{dt}\langle \log x(t)\rangle . \end{array}$$

This leads to a long run log return of 0, whereas on the short time scale the same observable behaves in the same way as sGBM. As such, the model can be used in order to model early herd behaviour, where the price of an asset grows simply as a consequence of investors following trends (short run behaviour), which last until the trade of the asset becomes congested (long run behaviour). The second log-moment is

(81) $$\begin{array}{cc} \langle \log^{2}x(t)\rangle & =\langle \log^{2}x_{0}\rangle +\left\{2\bar{\mu }\langle \log x_{0}\rangle +\sigma^{2}\right\}\tau^{\alpha }\frac{\gamma (\alpha ,t/\tau )}{\Gamma (\alpha )}+2{\bar{\mu }}^{2}\tau^{2\alpha }\frac{\gamma (2\alpha ,t/\tau )}{\Gamma (2\alpha )}, \end{array}$$

from where the log-variance becomes

(82) $$\begin{array}{cc} \langle \log^{2}x(t)\rangle -{\langle \log x(t)\rangle }^{2} & =\sigma^{2}\tau^{\alpha }\frac{\gamma (\alpha ,t/\tau )}{\Gamma (\alpha )}+{\bar{\mu }}^{2}\tau^{2\alpha }\left[2\frac{\gamma (2\alpha ,t/\tau )}{\Gamma (2\alpha )}-\frac{\gamma^{2}\left(\alpha ,t/\tau \right)}{\Gamma^{2}\left(\alpha \right)}\right]. \end{array}$$

Here, we note that, for t/τ≪1, the obtained results correspond to those that were obtained for sGBM, as it should be since the exponential truncation has no influence on the process. We observe that on the long run the log-variance becomes constant, i.e., it is equal to $\sigma^{2}\tau^{\alpha }+\bar{\mu }\;\tau^{2\alpha }$. In a similar way, for the third and fourth log-moments, we find

(83) $$\begin{array}{cc} \langle \log^{3}x(t)\rangle & =\langle \log^{3}x_{0}\rangle +3\left\{\bar{\mu }\langle \log^{2}x_{0}\rangle +\sigma^{2}\langle \log x_{0}\rangle \right\}\tau^{\alpha }\frac{\gamma (\alpha ,t/\tau )}{\Gamma (\alpha )}\\ & +6\bar{\mu }\left\{\bar{\mu }\langle \log x_{0}\rangle +\sigma^{2}\right\}\tau^{2\alpha }\frac{\gamma (2\alpha ,t/\tau )}{\Gamma (2\alpha )}+6{\bar{\mu }}^{3}\tau^{3\alpha }\frac{\gamma (3\alpha ,t/\tau )}{\Gamma (3\alpha )}, \end{array}$$

(84) $$\begin{array}{cc} \langle \log^{4}x(t)\rangle & =\langle \log^{4}x_{0}\rangle +2\left\{2\bar{\mu }\langle \log^{3}x_{0}\rangle +3\sigma^{2}\langle \log^{2}x_{0}\rangle \right\}\tau^{\alpha }\frac{\gamma (\alpha ,t/\tau )}{\Gamma (\alpha )}\\ & +6\left\{2\bar{\mu }\left(\bar{\mu }\langle \log^{2}x_{0}\rangle +2\sigma^{2}\langle \log x_{0}\rangle \right)+\sigma^{4}\right\}\tau^{2\alpha }\frac{\gamma (2\alpha ,t/\tau )}{\Gamma (2\alpha )}\\ & +24{\bar{\mu }}^{2}\left\{\bar{\mu }\langle \log x_{0}\rangle +3\sigma^{2}/2\right\}\tau^{3\alpha }\frac{\gamma (3\alpha ,t/\tau )}{\Gamma (3\alpha )}+24{\bar{\mu }}^{4}\tau^{4\alpha }\frac{\gamma (4\alpha ,t/\tau )}{\Gamma (4\alpha )}. \end{array}$$

The subordination function, in this case, is given by

(85) $$\begin{array}{cc} \; & \hat{h}\left(u,s\right)=\frac{{\left(s+\tau^{-1}\right)}^{\alpha }}{s}e^{-u{\left(s+\tau^{-1}\right)}^{\alpha }}=\left[1+{\left(s\tau \right)}^{-1}\right]{\left(s+\tau^{-1}\right)}^{\alpha -1}e^{-u{\left(s+\tau^{-1}\right)}^{\alpha }},\\ \; & h\left(u,t\right)=e^{-t/\tau }\;H_{1,1}^{1,0}\left[\frac{u}{t^{\alpha }}\left|\begin{array}{c} \left(1,\alpha \right)\\ \left(1,1\right) \end{array}\right.\right]+\tau^{-1}\int_{0}^{t}e^{-t^{\prime }/\tau }\;H_{1,1}^{1,0}\left[\frac{u}{t^{\prime \alpha }}\left|\begin{array}{c} \left(1,\alpha \right)\\ \left(1,1\right) \end{array}\right.\right]dt^{\prime }, \end{array}$$

from where one can analyse the PDF P(x,t).

### 3.5. Combined Standard and Subdiffusive GBM

As another application, let us consider the combination of GBM and sGBM, represented by the memory kernel

(86) $$\begin{array}{c} \eta \left(t\right)=w_{1}\frac{t^{\alpha -1}}{\Gamma (\alpha )}+w_{2}, \end{array}$$

where 0<α<1, $w_{1}+w_{2}=1$, and

(87) $$\begin{array}{c} \hat{\eta }\left(s\right)=w_{1}s^{-\alpha }+w_{2}s^{-1}. \end{array}$$

This case combines both motions governed by Equations (3) and (26). In this case, in a jump picture, normal GBM steps occur with weight $w_{2}$, while power-law waiting time steps are realised with weight $w_{1}$.

The mean value for this case is given by

(88) $$\begin{array}{cc} \langle x(t)\rangle & =x_{0}\;L^{-1}\left\{\frac{s^{-1}}{1-\mu \left(w_{1}s^{-\alpha }+w_{2}s^{-1}\right)}\right\}\left(t\right)=x_{0}\;\sum_{n=0}^{\infty }w_{1}^{n}\mu^{n}t^{\alpha n}E_{1,\alpha n+1}^{n+1}\left(w_{2}\mu t\right)\\ & =x_{0}\;\sum_{n=0}^{\infty }\frac{w_{1}^{n}\mu^{n}t^{\alpha n}}{\Gamma (\alpha n+1)}{}_{1}F_{1}\left(n+1;\alpha n+1;w_{2}\mu t\right), \end{array}$$

where ${}_{1}F_{1}\left(a;b;z\right)=\sum_{k=0}^{\infty }\frac{{\left(a\right)}_{k}}{{\left(b\right)}_{k}}\frac{z^{k}}{k!}$ is the Kummer confluent hypergeometric function,

(89) $$\begin{array}{c} E_{\alpha ,\beta }^{\gamma }\left(z\right)=\sum_{n=0}^{\infty }\frac{{\left(\gamma \right)}_{n}}{\Gamma (\alpha n+\beta )}\frac{z^{n}}{n!}, \end{array}$$

is the three parameter ML function [63], and ${\left(\gamma \right)}_{n}=\Gamma \left(\gamma +n\right)/\Gamma \left(\gamma \right)$ is the Pochhammer symbol. The Laplace transform of the three parameter ML function reads $L\left\{t^{\beta -1}E_{\alpha ,\beta }^{\gamma }\left(at^{\alpha }\right)\right\}\left(s\right)=\frac{s^{\alpha \gamma -\beta }}{{\left(s^{\alpha }-a\right)}^{\gamma }}$. From here, we see that, for $w_{1}=0$ and $w_{2}=1$, only the term for n=0 in Equation (88) survives, which yields the result for standard GBM as it should be. The opposite case, with $w_{1}=1$ and $w_{2}=0$, yields

(90) $$\begin{array}{cc} \langle x(t)\rangle & =x_{0}\;\sum_{n=0}^{\infty }\frac{\mu^{n}t^{\alpha n}}{\Gamma (\alpha n+1)}=E_{\alpha }\left(\mu t^{\alpha }\right), \end{array}$$

as it should be for the sGBM. For the second, third, and fourth moments, we find

(91) $$\begin{array}{c} \langle x^{2}\left(t\right)\rangle =x_{0}^{2}\;\sum_{n=0}^{\infty }w_{1}^{n}{\left(\sigma^{2}+2\mu \right)}^{n}t^{\alpha n}E_{1,\alpha n+1}^{n+1}\left(w_{2}\left(\sigma^{2}+2\mu \right)t\right), \end{array}$$

(92) $$\begin{array}{c} \langle x^{3}\left(t\right)\rangle =x_{0}^{3}\;\sum_{n=0}^{\infty }w_{1}^{n}{\left(3\sigma^{2}+3\mu \right)}^{n}t^{\alpha n}E_{1,\alpha n+1}^{n+1}\left(w_{2}\left(3\sigma^{2}+3\mu \right)t\right), \end{array}$$

(93) $$\begin{array}{c} \langle x^{4}\left(t\right)\rangle =x_{0}^{4}\;\sum_{n=0}^{\infty }w_{1}^{n}{\left(6\sigma^{2}+4\mu \right)}^{n}t^{\alpha n}E_{1,\alpha n+1}^{n+1}\left(w_{2}\left(6\sigma^{2}+4\mu \right)t\right). \end{array}$$

Following the same procedure as previously, for the log-mean we find

(94) $$\begin{array}{c} \langle \log x(t)\rangle =\langle \log x_{0}\rangle +\bar{\mu }\left[w_{1}\;\frac{t^{\alpha }}{\Gamma (\alpha +1)}+w_{2}\;t\right]. \end{array}$$

and for the expectation of the periodic log return with period Δt,

(95) $$\begin{array}{c} \frac{1}{\Delta t}\langle \log\left(x(t+\Delta t)/x(t)\right)\rangle \underset{\Delta t\to 0}{∼}\bar{\mu }\left[w_{1}\;\frac{t^{\alpha -1}}{\Gamma (\alpha )}+w_{2}\right]=\frac{d}{dt}\langle \log x(t)\rangle . \end{array}$$

This model introduces subdiffusive and trapping asset dynamics on short time scales (i.e., then the part multiplied with $w_{1}$ is much bigger), whereas, on the long run, we recover the standard GBM dynamics. The second log-moment yields

(96) $$\begin{array}{cc} \langle \log^{2}x(t)\rangle & =\langle \log^{2}x_{0}\rangle +\left\{2\bar{\mu }\langle \log x_{0}\rangle +\sigma^{2}\right\}\left[w_{1}\;\frac{t^{\alpha }}{\Gamma (\alpha +1)}+w_{2}\;t\right]\\ & +2{\bar{\mu }}^{2}\left[\frac{w_{1}^{2}t^{2\alpha }}{\Gamma (2\alpha +1)}+\frac{2w_{1}w_{2}t^{\alpha +1}}{\Gamma (\alpha +2)}+\frac{w_{2}^{2}t^{2}}{2}\right], \end{array}$$

from where the log-variance becomes

(97) $$\begin{array}{cc} \langle \log^{2}x(t)\rangle -{\langle \log x(t)\rangle }^{2} & =\sigma^{2}\left[w_{1}\;\frac{t^{\alpha }}{\Gamma (\alpha +1)}+w_{2}\;t\right]+{\bar{\mu }}^{2}w_{1}^{2}t^{2\alpha }\left[\frac{2}{\Gamma (2\alpha +1)}-\frac{1}{\Gamma^{2}\left(\alpha +1\right)}\right]\\ & +2{\bar{\mu }}^{2}w_{1}w_{2}t^{\alpha +1}\left[\frac{2}{\Gamma (\alpha +2)}-\frac{1}{\Gamma (\alpha +1)}\right]. \end{array}$$

Similarly to the behaviour of the first log moment, in the log variance, for short time scales, the sGBM dynamics dominates. However, we observe that, on the long run, the dynamics is a combination of the two kernels, since the dominant term is $w_{1}w_{2}t^{\alpha +1}$. Moreover, the third and fourth log-moments read

(98) $$\begin{array}{cc} \langle \log^{3}x(t)\rangle & =\langle \log^{3}x_{0}\rangle +3\left\{\bar{\mu }\langle \log^{2}x_{0}\rangle +\sigma^{2}\langle \log x_{0}\rangle \right\}\left(w_{1}\frac{t^{\alpha }}{\Gamma (\alpha +1)}+w_{2}t\right)\\ & +6\bar{\mu }\left\{\bar{\mu }\langle \log x_{0}\rangle +\sigma^{2}\right\}\left[\frac{w_{1}^{2}t^{2\alpha }}{\Gamma (2\alpha +1)}+\frac{2w_{1}w_{2}t^{\alpha +1}}{\Gamma (\alpha +2)}+\frac{w_{2}^{2}t^{2}}{2}\right]\\ & +6{\bar{\mu }}^{3}\left[\frac{w_{1}^{3}t^{3\alpha }}{\Gamma (3\alpha +1)}+\frac{3w_{1}^{2}w_{2}t^{2\alpha +1}}{\Gamma (2\alpha +2)}+\frac{3w_{1}w_{2}^{2}t^{\alpha +2}}{\Gamma (\alpha +3)}+\frac{w_{2}^{3}t^{3}}{6}\right], \end{array}$$

(99) $$\begin{array}{cc} \langle \log^{4}x(t)\rangle & =\langle \log^{4}x_{0}\rangle +2\left\{2\bar{\mu }\langle \log^{3}x_{0}\rangle +3\sigma^{2}\langle \log^{2}x_{0}\rangle \right\}\left[\frac{w_{1}t^{\alpha }}{\Gamma (\alpha +1)}+w_{2}t\right]\\ & +6\left\{2\bar{\mu }\left(\bar{\mu }\langle \log^{2}x_{0}\rangle +2\sigma^{2}\langle \log x_{0}\rangle \right)+\sigma^{4}\right\}\left[\frac{w_{1}^{2}t^{2\alpha }}{\Gamma (2\alpha +1)}+\frac{2w_{1}w_{2}t^{\alpha +1}}{\Gamma (\alpha +2)}+\frac{w_{2}^{2}t^{2}}{2}\right]\\ & +24{\bar{\mu }}^{2}\left\{\bar{\mu }\langle \log x_{0}\rangle +3\sigma^{2}/2\right\}\left[\frac{w_{1}^{3}t^{3\alpha }}{\Gamma (3\alpha +1)}+\frac{3w_{1}^{2}w_{2}t^{2\alpha +1}}{\Gamma (2\alpha +2)}+\frac{3w_{1}w_{2}^{2}t^{\alpha +2}}{\Gamma (\alpha +3)}+\frac{w_{2}^{3}t^{3}}{6}\right]\\ & +24{\bar{\mu }}^{4}\left[\frac{w_{1}^{4}t^{4\alpha }}{\Gamma (4\alpha +1)}+\frac{4w_{1}^{3}w_{2}t^{3\alpha +1}}{\Gamma (3\alpha +2)}+\frac{6w_{1}^{2}w_{2}^{2}t^{2\alpha +2}}{\Gamma (2\alpha +2)}+\frac{4w_{1}w_{2}^{3}t^{\alpha +3}}{\Gamma (\alpha +4)}+\frac{w_{2}^{4}t^{4}}{24}\right]. \end{array}$$

The subordination function for this case is given by

(100) $$\begin{array}{c} \hat{h}\left(u,s\right)=\frac{1}{w_{1}+w_{2}s^{1-\alpha }}e^{-\frac{u}{w_{1}s^{-1}+w_{2}s^{-\alpha }}}, \end{array}$$

where the Lévy exponent is $\hat{\Psi }\left(s\right)={\left[w_{1}s^{-1}+w_{2}s^{-\alpha }\right]}^{-1}$.

### 3.6. Mix of Subdiffusive GBMs

We may further analyse the case of a mix of two sGBM with different power-law memory functions,

(101) $$\begin{array}{c} \eta \left(t\right)=w_{1}\frac{t^{\alpha_{1}-1}}{\Gamma (\alpha_{1})}+w_{2}\frac{t^{\alpha_{2}-1}}{\Gamma (\alpha_{2})}, \end{array}$$

where $0<\alpha_{1}<\alpha_{2}<1$, $w_{1}+w_{2}=1$, and

(102) $$\begin{array}{c} \hat{\eta }\left(s\right)=w_{1}s^{-\alpha_{1}}+w_{2}s^{-\alpha_{2}}. \end{array}$$

This situation corresponds to the case of two different groups of periods of constant prices. For physical systems, this situation means that the process is a sum of two random processes with different waiting times [64], as represented by the memory kernel (101).

Therefore, for the mean, we find

(103) $$\begin{array}{cc} \langle x(t)\rangle & =x_{0}\;L^{-1}\left\{\frac{s^{-1}}{1-\mu \left(w_{1}s^{-\alpha_{1}}+w_{2}s^{-\alpha_{2}}\right)}\right\}\left(t\right)=x_{0}\;\sum_{n=0}^{\infty }w_{1}^{n}\mu^{n}t^{\alpha_{1}n}E_{\alpha_{2},\alpha_{1}n+1}^{n+1}\left(w_{2}\mu t^{\alpha_{2}}\right), \end{array}$$

while, for the second, third, and fourth moments, respectively, we obtain

(104) $$\begin{array}{c} \langle x^{2}\left(t\right)\rangle =x_{0}^{2}\;\sum_{n=0}^{\infty }w_{1}^{n}{\left(\sigma^{2}+2\mu \right)}^{n}t^{\alpha_{1}n}E_{\alpha_{2},\alpha_{1}n+1}^{n+1}\left(w_{2}\left(\sigma^{2}+2\mu \right)t^{\alpha_{2}}\right), \end{array}$$

(105) $$\begin{array}{c} \langle x^{3}\left(t\right)\rangle =x_{0}^{3}\;\sum_{n=0}^{\infty }w_{1}^{n}{\left(3\sigma^{2}+3\mu \right)}^{n}t^{\alpha_{1}n}E_{\alpha_{2},\alpha_{1}n+1}^{n+1}\left(w_{2}\left(3\sigma^{2}+3\mu \right)t^{\alpha_{2}}\right), \end{array}$$

(106) $$\begin{array}{c} \langle x^{4}\left(t\right)\rangle =x_{0}^{4}\;\sum_{n=0}^{\infty }w_{1}^{n}{\left(6\sigma^{2}+4\mu \right)}^{n}t^{\alpha_{1}n}E_{\alpha_{2},\alpha_{1}n+1}^{n+1}\left(w_{2}\left(6\sigma^{2}+4\mu \right)t^{\alpha_{2}}\right). \end{array}$$

Similarly, the log-mean yields

(107) $$\begin{array}{c} \langle \log x(t)\rangle =\langle \log x_{0}\rangle +\bar{\mu }\left[w_{1}\;\frac{t^{\alpha_{1}}}{\Gamma (\alpha_{1}+1)}+w_{2}\;\frac{t^{\alpha_{2}}}{\Gamma (\alpha_{2}+1)}\right]. \end{array}$$

The expectation of the log return with period Δt, then becomes

(108) $$\begin{array}{c} \frac{1}{\Delta t}\langle \log\left(x(t+\Delta t)/x(t)\right)\rangle \underset{\Delta t\to 0}{∼}\bar{\mu }\left[w_{1}\;\frac{t^{\alpha_{1}-1}}{\Gamma (\alpha_{1})}+w_{2}\;\frac{t^{\alpha_{2}-1}}{\Gamma (\alpha_{2})}\right]=\frac{d}{dt}\langle \log x(t)\rangle . \end{array}$$

Because $0<\alpha_{1}<\alpha_{2}<1$, on short times, the part of first sGBM dominates, whereas, on long times, it is the characteristic of the second sGBM that determines the dynamics. The second log-moment becomes

(109) $$\begin{array}{cc} \langle \log^{2}x(t)\rangle & =\langle \log^{2}x_{0}\rangle +\left\{2\bar{\mu }\langle \log x_{0}\rangle +\sigma^{2}\right\}\left[w_{1}\frac{t^{\alpha_{1}}}{\Gamma (\alpha_{1}+1)}+w_{2}\frac{t^{\alpha_{2}}}{\Gamma (\alpha_{2}+1)}\right]\\ & +2{\bar{\mu }}^{2}\left[\frac{w_{1}^{2}t^{2\alpha_{1}}}{\Gamma (2\alpha_{1}+1)}+\frac{2w_{1}w_{2}t^{\alpha_{1}+\alpha_{2}}}{\Gamma (\alpha_{1}+\alpha_{2}+1)}+\frac{w_{2}^{2}t^{2\alpha_{2}}}{\Gamma (2\alpha_{2}+1)}\right], \end{array}$$

and for the log-variance we find

(110) $$\begin{array}{cc} \langle \log^{2}x(t)\rangle -{\langle \log x(t)\rangle }^{2} & =\sigma^{2}\left[w_{1}\frac{t^{\alpha_{1}}}{\Gamma (\alpha_{1}+1)}+w_{2}\frac{t^{\alpha_{2}}}{\Gamma (\alpha_{2}+1)}\right]\\ & +2{\bar{\mu }}^{2}w_{1}w_{2}t^{\alpha_{1}+\alpha_{2}}\left[\frac{2}{\Gamma (\alpha_{1}+\alpha_{2}+1)}-\frac{1}{\Gamma \left(\alpha_{1}+1\right)\Gamma \left(\alpha_{2}+1\right)}\right]\\ & +{\bar{\mu }}^{2}w_{1}^{2}t^{2\alpha_{1}}\left[\frac{2}{\Gamma (2\alpha_{1}+1)}-\frac{1}{\Gamma^{2}\left(\alpha_{1}+1\right)}\right]\\ & +{\bar{\mu }}^{2}w_{2}^{2}t^{2\alpha_{2}}\left[\frac{2}{\Gamma (2\alpha_{2}+1)}-\frac{1}{\Gamma^{2}\left(\alpha_{2}+1\right)}\right]. \end{array}$$

In this case, for short times, the kernel with the smaller exponent dominates the variance. Interestingly, for long times, this observable is determined by the magnitude of the larger exponent, which is opposite from the previous kernel examples. Moreover, the third and fourth log-moments read

(111) $$\begin{array}{cc} \langle \log^{3}x(t)\rangle & =\langle \log^{3}x_{0}\rangle +3\left\{\bar{\mu }\langle \log^{2}x_{0}\rangle +\sigma^{2}\langle \log x_{0}\rangle \right\}\left(w_{1}\frac{t^{\alpha_{1}}}{\Gamma (\alpha_{1}+1)}+w_{2}\frac{t^{\alpha_{2}}}{\Gamma (\alpha_{2}+1)}\right)\\ & +6\bar{\mu }\left\{\bar{\mu }\langle \log x_{0}\rangle +\sigma^{2}\right\}\left(w_{1}^{2}\frac{t^{2\alpha_{1}}}{\Gamma (2\alpha_{1}+1)}+2w_{1}w_{2}\frac{t^{\alpha_{1}+\alpha_{2}}}{\Gamma (\alpha_{1}+\alpha_{2}+1)}+w_{2}^{2}\frac{t^{2\alpha_{2}}}{\Gamma (2\alpha_{2}+1)}\right)\\ & +6{\bar{\mu }}^{3}\left[\frac{w_{1}^{3}t^{3\alpha_{1}}}{\Gamma (3\alpha_{1}+1)}+\frac{3w_{1}^{2}w_{2}t^{2\alpha_{1}+\alpha_{2}}}{\Gamma (2\alpha_{1}+\alpha_{2}+1)}+\frac{3w_{1}w_{2}^{2}t^{\alpha_{1}+2\alpha_{2}}}{\Gamma (\alpha_{1}+2\alpha_{2}+1)}+\frac{w_{2}^{3}t^{3\alpha_{2}}}{\Gamma (3\alpha_{2}+1)}\right], \end{array}$$

(112) $$\begin{array}{cc} \; & \langle \log^{4}x(t)\rangle =\langle \log^{4}x_{0}\rangle +2\left\{2\bar{\mu }\langle \log^{3}x_{0}\rangle +3\sigma^{2}\langle \log^{2}x_{0}\rangle \right\}\left(\frac{w_{1}t^{\alpha_{1}}}{\Gamma (\alpha_{1}+1)}+\frac{w_{2}t^{\alpha_{2}}}{\Gamma (\alpha_{2}+1)}\right)\\ & +6\left\{2\bar{\mu }\left(\bar{\mu }\langle \log^{2}x_{0}\rangle +2\sigma^{2}\langle \log x_{0}\rangle \right)+\sigma^{4}\right\}\left(\frac{w_{1}^{2}t^{2\alpha_{1}}}{\Gamma (2\alpha_{1}+1)}+\frac{2w_{1}w_{2}t^{\alpha_{1}+\alpha_{2}}}{\Gamma (\alpha_{1}+\alpha_{2}+1)}+\frac{w_{2}^{2}t^{2\alpha_{2}}}{\Gamma (2\alpha_{2}+1)}\right)\\ & +24{\bar{\mu }}^{2}\left\{\bar{\mu }\langle \log x_{0}\rangle +3\sigma^{2}/2\right\}\left[\frac{w_{1}^{3}t^{3\alpha_{1}}}{\Gamma (3\alpha_{1}+1)}+\frac{3w_{1}^{2}w_{2}t^{2\alpha_{1}+\alpha_{2}}}{\Gamma (2\alpha_{1}+\alpha_{2}+1)}+\frac{3w_{1}w_{2}^{2}t^{\alpha_{1}+2\alpha_{2}}}{\Gamma (\alpha_{1}+2\alpha_{2}+1)}+\frac{w_{2}^{3}t^{3\alpha_{2}}}{\Gamma (3\alpha_{2}+1)}\right]\\ & +24{\bar{\mu }}^{4}\left[\frac{w_{1}^{4}t^{4\alpha_{1}}}{\Gamma (4\alpha_{1}+1)}+\frac{4w_{1}^{3}w_{2}t^{3\alpha_{1}+\alpha_{2}}}{\Gamma (3\alpha_{1}+\alpha_{2}+1)}+\frac{6w_{1}^{2}w_{2}^{2}t^{2\alpha_{1}+2\alpha_{2}}}{\Gamma (2\alpha_{1}+2\alpha_{2}+1)}+\frac{4w_{1}w_{2}^{3}t^{\alpha_{1}+3\alpha_{2}}}{\Gamma (\alpha_{1}+3\alpha_{2}+1)}+\frac{w_{2}^{4}t^{4\alpha_{2}}}{\Gamma (4\alpha_{2}+1)}\right]. \end{array}$$

For the mix of subdiffusive GBMs, the subordination function becomes

(113) $$\begin{array}{c} \hat{h}\left(u,s\right)=\frac{1}{w_{1}s^{1-\alpha_{1}}+w_{2}s^{1-\alpha_{2}}}e^{-\frac{u}{w_{1}s^{-\alpha_{1}}+w_{2}s^{-\alpha_{2}}}}, \end{array}$$

where the Lévy exponent is $\hat{\Psi }\left(s\right)={\left[w_{1}s^{-\alpha_{1}}+w_{2}s^{-\alpha_{2}}\right]}^{-1}$, see Refs. [64,65].

## 4. Numerical Experiments

**Figure 1:**
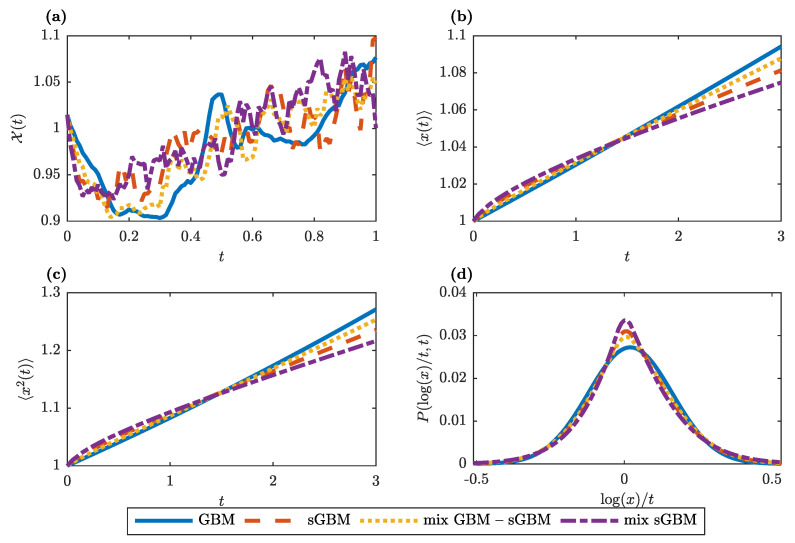
Generalised geometric Brownian motion (gGBM) properties. (**a**) An example for simulated individual trajectories of gGBM for different memory kernels: standard GBM (blue solid line), subdiffusive GBM (sGBM) (red dashed line), a mix of standard GBM and sGBM (yellow dotted line), and a mix of sGBM (violet dot-dashed line). (**b**) Numerical estimation for the first moment in GBM, sGBM, a mix of standard GBM and sGBM and a mix of sGBM as a function of time. (**c**) Same as (**b**), only for the second moment. (**d**) Empirical PDF for the logarithmic return at t=1 year estimated from 1000 realisations of gGBM. (**a**–**d**) In the simulations, μ=0.03 and $\sigma^{2}=0.02$. Moreover, for the sGBM case we set α=0.8, for the mix GBM-sGBM case, we set α=0.8 and $w_{1}=w_{2}=0.5$, and for the mix of sGBM case $\alpha_{1}=0.8$, $\alpha_{2}=0.6$, and $w_{1}=w_{2}=0.5$.

Figure 1a provides an intuitive illustration of the gGBM dynamics under various choices for the kernel. As argued, for standard GBM we observe smooth dynamics without periods of constant prices, whereas there is more turbulence in the asset price dynamics in the gGBM case. The periods of constant prices that are reproduced by gGBM depend, in general, on the time scale and, hence, the measuring units of the drift and volatility, with longer time scales also corresponding to longer periods of constant prices. In Figure 1b,c we plot, respectively, the numerical approximations for the first moment and the MSD for GBM, sGBM, a mix of GBM and sGBM, and a mix of sGBMs. One can easily notice the nonlinear behaviour in the generalisations of GBM. For long times, all gGBMs give an exponential dependence of the first moment and the MSD on time but with smaller slope than the one of GBM. Finally, Figure 1d shows the empirical PDF for the logarithmic return at t=1 year. For each of the studied generalisations of GBM, the PDF is characterised with fatter tails (which should increase as the α parameters decrease), which means that it is more prone to producing values that fall far from the average.

**Figure 2:**
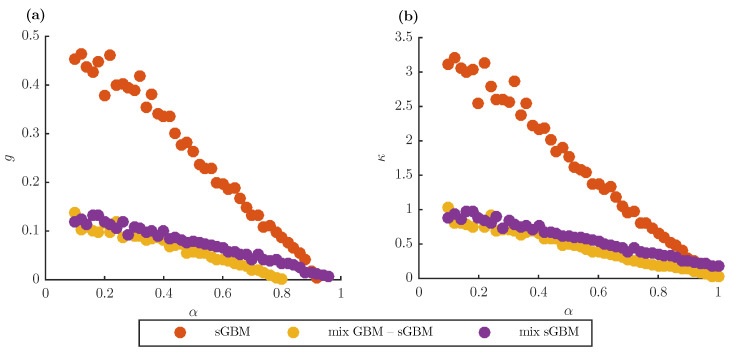
gGBM skewness and excess kurtosis. (**a**) Skewness for the distribution of logarithmic return for sGBM, a mix of standard GBM and sGBM and a mix of sGBM at t=1 year as a function of α (for sGBM and the mix of GBM-sGBM) or $\alpha_{1}$ (for the mix of sGBMs). (**b**) Same as (**a**), only for the excess kurtosis. (**a**,**b**) The PDF for the logarithmic return at t=1 year is estimated from 1000 realisations of gGBM. In the simulations, μ=0.03 and $\sigma^{2}=0.02$. Moreover, for the mix GBM-sGBM case, we set $w_{1}=w_{2}=0.5$, and for the mix of sGBM case $\alpha_{2}=0.8$ and $w_{1}=w_{2}=0.5$.

The fat-tailed property can be observed in greater detail in Figure 2, where we plot the skewness *g* and excess kurtosis κ in gGBM as a function of α (for sGBM and the mix of GBM-sGBM) or $\alpha_{1}$ (for the mix of sGBM) for the logarithmic return at t=1 year. As is the case with general fat-tailed distributions, all of the generalisations exhibit positive skewness and excess kurtosis. This is exactly what makes the gGBM framework useful in understanding the statistical behaviour of the asset price dynamics. Moreover, the figure indicates that there is an inverse relationship between these two statistics and α ($\alpha_{1}$), i.e., as α ($\alpha_{1}$) increases, *g* and κ decrease. For small α ($\alpha_{1}$), sGBM is the model with the largest skewness and excess kurtosis, followed by the mix of sGBM and the mix of GBM-sGBM. For large α ($\alpha_{1}$), the mix of GBM-sGBM remains the model with the lowest *g* and κ, but, now, the mix of sGBM becomes the model with the largest values of the two statistics. The rationale for this observation is that the GBM-sGBM model already includes a GBM term, which greatly induces the fat tails, whereas the operational time in the mix of sGBM becomes dominated by the value of the second sGBM, which has a fixed value for $\alpha_{2}$, thereby resulting in larger skewness and excess kurtosis.

Finally, we investigate the dependence of two standard quantities that are relevant in an option pricing scheme: (i) at-the-money (ATM) implied volatility and (ii) ATM volatility skew with respect to the gGBM parameters. Both of the quantities are a part of the moneyness property of the option. Moneyness describes the relative position of the current price of the asset ($x_{0}$) with respect to the strike price of the option (*K*). An option whose strike price is equal to the current price of the asset is said to be at-the-money; if the strike price is larger than the current price, the option is “out of the money”; and, if the strike price is smaller than the current price, the option is described to be “in the money”.

Moneyness is usually examined by plotting the implied volatility of an option for an underlying asset as a function of its strike price. This plot is formally known as the volatility smile. Its name is derived from the usual pattern, which suggests that the implied volatility is the lowest for options that are ATM, i.e., the plot looks like a parabola (smile). The ATM volatility skew is the first derivative of the volatility smile at the ATM point [66]. A larger volatility skew implies that the implied volatility increases faster for options that are near the money (options whose strike price is near the current asset price), and vice versa.

**Figure 3:**
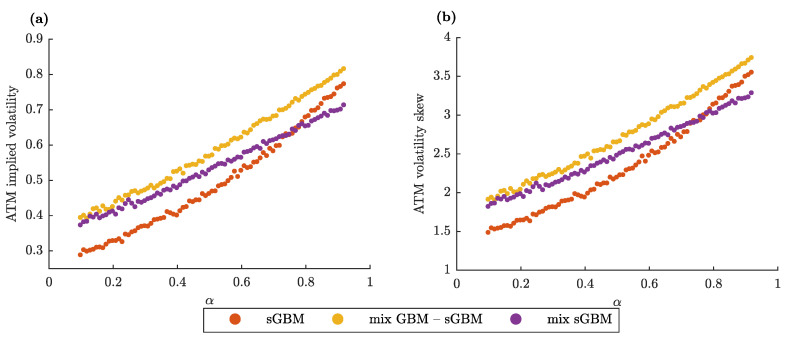
gGBM at-the-money (ATM) implied volatility and volatility skew. (**a**) ATM implied volatility for sGBM, a mix of standard GBM and sGBM and a mix of sGBM for an option with $K=x_{0}=1$, T=0.083 years (one month) as a function of α (for sGBM and the mix of GBM-sGBM) or $\alpha_{1}$ (for the mix of sGBMs). (**b**) Same as (**a**), only for the ATM volatility skew. (**a**,**b**) We assume that r=0.02. Moreover, for the mix GBM-sGBM case, we set $w_{1}=w_{2}=0.5$, and for the mix of sGBM case $\alpha_{2}=0.8$ and $w_{1}=w_{2}=0.5$.

Figure 3 displays the functional relationship between the two studied quantities and the parameter α (for sGBM and the mix of GBM-sGBM) or $\alpha_{1}$ (for the mix of sGBMs). For each gGBM model, we get that lower ATM implied volatilities can be attributed to lower diffusion rates, thereby suggesting that the gGBM framework can be used to describe the empirical at-the-money observations. Identically, there is a direct relationship between ATM volatility skews and α ($\alpha_{1}$). In other words, in the gGBM framework, the slope of the volatility smile for at-the-money options behaves in the same manner as the magnitude of the implied volatility. This leads to lower near-the-money implied volatilities.

## 5. Empirical Example

In order to illustrate the power of the gGBM framework in the evaluation of options, we utilise empirical data of American options for two companies: Tesla (TSLA) and Apple (AAPL). By definition, the dynamics of American options differ from European, as they allow for exercising of the option at any time before the option expires. Nevertheless, as given in Ref. [67], one can rely on the fact that American options on non-dividend-paying stocks have the same value as their European counterpart. This relation has allowed for the empirical examination of a pricing scheme of European options to be widely done via data for American ones.

For our analysis, we use freely available data from the Nasdaq’s Options Trading Centre. This dataset offers daily data for options of all companies quoted on the NASDAQ stock market. However, the options for most companies have small sample size. Therefore, we restricted the empirical analysis to Tesla and Apple, whose options are more frequently traded. In our estimations, the risk-free rate of return *r* is taken simply as the three-Month Treasury Bill Secondary Market Rate at the date of observation. The volatility parameter σ, on the other hand, was inferred from the values of the options on the market as the value that produces the minimum root mean squared pricing error (RMSE) in their fit.

For both assets, we evaluate the predictive performance of the same sGBM, a mix of GBM-sGBM, and a mix of sGBM models that were used in the numerical analysis of the previous section. The predicted option prices by the model were inferred via a Monte Carlo estimation of (59) (see Refs [28,48]).

**Figure 4:**
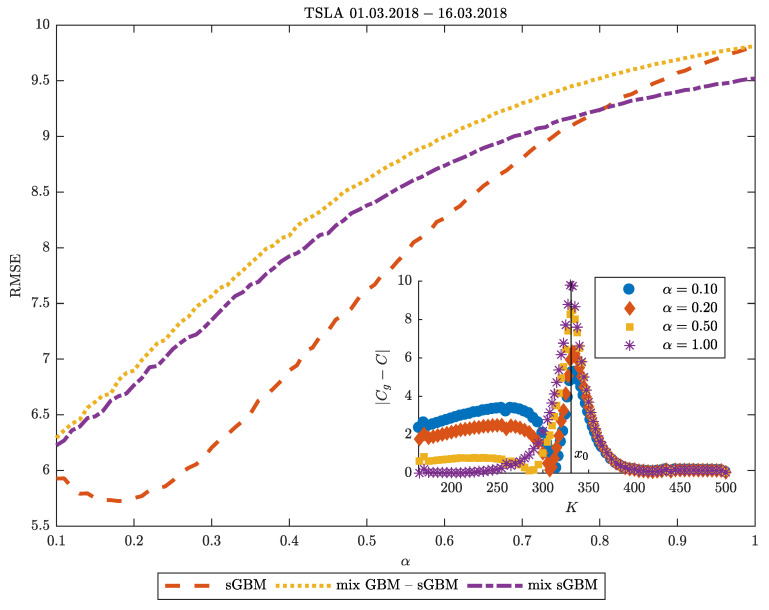
Moneyness in Tesla (TSLA). Root mean squared error (RMSE) of the predicted option price as a function of α ($\alpha_{1}$) for sGBM, a mix of GBM-sGBM and a mix of sGBM. The inset plot gives the difference between the predicted TSLA option price $C_{g}$ and its real value *C* as a function of the strike price of the option for various choices of α. The data is taken on 1st March 2020 and describe the value of TSLA options that expire on 16th March 2020. For the mix GBM-sGBM case we set $w_{1}=w_{2}=0.5$, and for the mix of sGBM case $\alpha_{2}=0.8$ and $w_{1}=w_{2}=0.5$.

**Moneyness in TSLA:** Let us now turn our attention to Figure 4, where we use TSLA data gathered on 1st March 2018 on options which expire on 16th March 2018 to examine the dependence of the sGBM, the mix of GBM-sGBM and the mix of sGBM models on α in predicting the option price. We discover that, in general, the TSLA asset price dynamics are best described as a sGBM, and the minimal error occurs around α=0.2. For large α, the mix of sGBM becomes the best performer, because it inherently includes a process with a lower subdiffusion and, thus, it has a close resemblance to the sGBM process with low α than the other models. For every α, the mix of GBM-sGBM has the worst performance since it includes a term with a normal diffusion.

In the inset plot of Figure 4, we provide a more detailed information on the predictive properties of sGBM by examining its relation with the moneyness of the option. In it, we vary the diffusion parameter α, and plot the absolute difference in the estimated option price $C_{g}$ and the observed option price as a function of the strike price. We find that, for in-the-money-options, the best prediction is with α=1, which corresponds to the BS model. However, as the strike price of the option nears the TSLA price, a transition occurs and α=1 becomes the worst predictor of the option price, whereas the lower the subdiffusion parameter, the better prediction we obtain. For options that are out of the money, it appears that the performance of the prediction for the option price does not depend on α. These findings are in agreement with the discussion presented in the previous section regarding the ATM implied volatility and ATM volatility skew.

**Maturity in Apple (AAPL):** Next, we use AAPL data that were gathered for at-the-money options on 28th February 2018 and examine how the maturity *T* affects the performance of the same models in predicting the option price. We focus solely on data for at-the-money options in order to remove the potential bias in the prediction error that arises from the potential moneyness effect, which as we saw in the above paragraph might arise (i.e., we discovered that the error rate with respect to α ($\alpha_{1}$) depends on the moneyness of the option).

For this purpose, in Table 1, we show the minimum RMSE and the corresponding optimal α ($\alpha_{1}$) of the AAPL option price prediction for seven different maturity periods. We observe that for the shortest-term maturity (T=0.006 years, or two days), a subdiffusive model (in particular sGBM or a mix of GBM-sGBM) is the model that best describes the option price. As the maturity increases, the optimal α ($\alpha_{1}$) also increases, reaching a maximal value of 1 for options maturing at T=0.101 years (one month) and T=0.14 years (35 working days). However, for the options with the longest maturity, again a model with a very low subdiffusion rate is an optimal fit. Hence, the RMSE of the prediction depends on both the maturity of the options and choice of α ($\alpha_{1}$).

**Figure 5:**
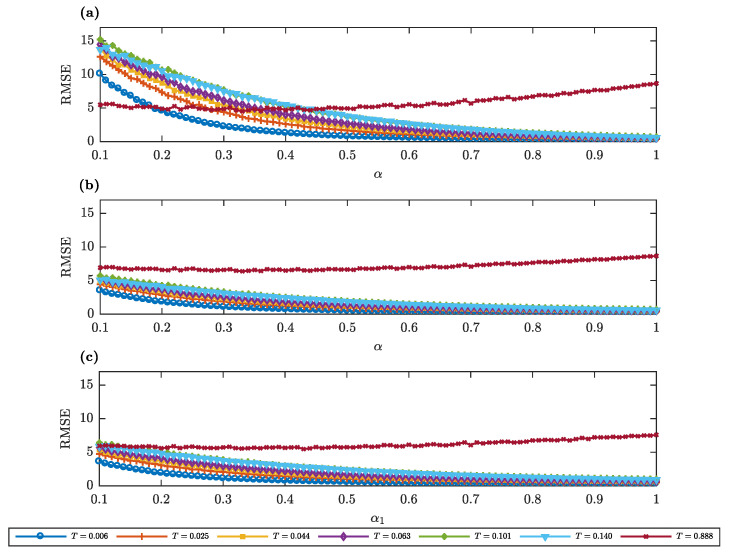
Maturity in at-the-money AAPL options. Root mean squared error (RMSE) of the prediction of the AAPL at-the-money option price with data taken on 28th February 2018 as a function of α for various maturity periods *T* (measured in years). (**a**) The gGBM model is sGBM. (**b**) The gGBM model is a mix of GBM-sGBM. (**c**) The gGBM model is a mix of sGBM. For the mix GBM-sGBM case we set $w_{1}=w_{2}=0.5$, and for the mix of sGBM case $\alpha_{2}=0.8$ and $w_{1}=w_{2}=0.5$.

To provide a better depiction on the role of α ($\alpha_{1}$), in the predictive performance of sGBM, a mix of GBM-sGBM and a mix of sGBM, in Figure 5 we depict the RMSE of the option price prediction as a function of the parameter α ($\alpha_{1}$). We observe that, in general, only for the options with the longest maturity, one can observe a clear extremum, whereas, for every other maturity, it appears that every α ($\alpha_{1}$) represents an adequate fit. Increasing this parameter will only lead to marginal and insignificant improvements in the error rate. As a consequence, one might even argue that different gGBM kernels can lead to similar outcomes in the pricing of options, an interesting finding as such.

Evidently, the performance of a kernel ultimately depends on the physical properties of the option. On the first sight, this conclusion appears intuitive—obviously the known information for the properties of the asset greatly impacts its price, the observation that a slight change in the known information may drastically change the dynamics suggests that there is a need in the option pricing literature for models that easily allow for such structural changes. In this aspect, we believe that the generalised GBM approach offers a computationally inexpensive and efficiently tractable solution to this issue. Consequently, we stress that a significant improvement of the description of the data in the gGBM framework can be achieved with comparatively few additional parameters.

## 6. Conclusions

We investigated the potential of GBM extensions that are based on subdiffusion to model and predict the price of options. By assuming that the price of the asset underlying the option undergoes a subdiffusive process, we introduced the gGBM framework as a potential model for its value.

Similar to previous works on subdiffusive GBM models, the dynamics of a particular gGBM instance is critically determined by a memory kernel. The advantage of gGBM comes in the flavour of allowing various forms for the functional form of the kernel. Depending on its choice, we may end up with asset price dynamics whose behaviour significantly varies on the short time in comparison to its long run characteristics. This, in turn, may induce observations of the properties of the asset price that more closely mimic realistic behaviour than standard GBM.

We explored the ability of gGBM to fit and predict real option values. Our empirical analysis confirmed the characteristics of gGBM, as we discovered that the performance of a certain choice of memory kernel is uniquely determined by the parameters of the option, such as its maturity and its moneyness. Because each kernel produces, in general, different long run and short run dynamics, this suggests that time-averages play an important role in efficient pricing of options. Formally, time-averaging is essential in the analysis of a single time-series (or a set of few), which is characterised with non-ergodic dynamics. The non-ergodicity creates non-equilibrium dynamics which, consequently, makes studies of the ensemble behaviour irrelevant. This leads to the introduction of novel strategies for analysing financial data [9,68].

In line with our conclusions, we believe that the next step in uncovering the properties of gGBM is demonstrating the ergodicity breaking of the process. Because multiplicative processes are frequently present in nature, this will not only extend the framework of gGBM in analysing financial data, but will also provide an avenue for applying the model in other scientific domains. Another fruitful research direction would be to incorporate the properties of gGBM in a wider framework for financial modelling, which includes the concept of “rough volatility”, where the instantaneous volatility is driven by a (rough) fractional Brownian motion [69]. Building an explanatory model for the volatility in terms of gGBM would bring novel insights regarding the theoretical and empirical characteristics of the asset prices. We also leave for future analysis the problem of gGBM with stochastic volatility, which can be treated in the framework of the Fokker–Planck equation for gGBM with time varying volatility σ(t), in analogy of the diffusing-diffusivity models for heterogeneous media [56,70,71,72,73].

## Figures and Tables

**Table 1:** Minimum prediction error and optimal α ($\alpha_{1}$) for ATM AAPL options. For the mix GBM-sGBM case we set $w_{1}=w_{2}=0.5$, and for the mix of sGBM case $\alpha_{2}=0.8$ and $w_{1}=w_{2}=0.5$.

Maturity	sGBM		GBM-sGBM		mix of sGBM	
(in Years)	minα	RMSE	minα	RMSE	$\min\alpha_{1}$	RMSE
0.006	0.89	0.28	0.84	0.28	0.88	0.31
0.025	0.96	0.39	0.93	0.39	0.93	0.48
0.044	0.97	0.46	0.93	0.46	0.97	0.6
0.063	0.99	0.52	0.98	0.52	0.98	0.71
0.101	1.00	0.72	0.99	0.72	0.99	1.00
0.140	1.00	0.61	0.99	0.60	0.99	0.91
0.888	0.33	4.52	0.35	6.38	0.38	5.47

## Abbreviations

The following abbreviations are used in this manuscript:

GBM	Geometric Brownian motion
sGBM	Subdiffusive geometric Brownian motion
gGBM	Generalised geometric Brownian motion
BS	Black-Scholes
CTRW	Continuous time random walk
MSD	Mean squared displacement
ML	Mittag-Leffler
ATM	at-the-money
RMSE	Root mean squared error
TSLA	Tesla
AAPL	Apple

## Appendix A. Solution of the Fokker-Planck Equation for Standard GBM

The solution of Equation (3) can be found by using the Laplace-Mellin transform method [74]. The Laplace transform is defined by

$$\hat{F}\left(s\right)=L\left\{f(t)\right\}\left(s\right)=\int_{0}^{\infty }f\left(t\right)\;e^{-st}\;dt,$$

while the Mellin transform as [75]

$$\tilde{F}\left(q\right)=M\left\{f(x)\right\}\left(q\right)=\int_{0}^{\infty }x^{q-1}\;f\left(x\right)\;dx.$$

The inverse Mellin transform then reads

$$f\left(x\right)=M^{-1}\left\{\tilde{F}\left(q\right)\right\}\left(x\right)=\frac{1}{2\pi ı}\int_{c-ı\infty }^{c+ı\infty }x^{-q}\;\tilde{F}\left(q\right)\;dq.$$

Therefore, by performing Laplace transform in respect to *t* and Mellin transform in respect to *x* in Equation (3), we have

(A1) $$\begin{array}{c} \tilde{\hat{F}}\left(q,s\right)=x_{0}^{q-1}\times \frac{1}{s-\left[\frac{\sigma^{2}}{2}\left(q-1\right)\left(q-2\right)+\mu \left(q-1\right)\right]}, \end{array}$$

where we use $M\left\{\delta (x-x_{0})\right\}\left(q\right)=x_{0}^{q-1}$. Then the inverse Laplace transform yields

(A2) $$\begin{array}{cc} \tilde{F}\left(q,t\right) & =x_{0}^{q-1}\times \exp\left(\frac{\sigma^{2}}{2}{\left[q+\frac{1}{2}\left(\frac{2\mu }{\sigma^{2}}-3\right)\right]}^{2}t-\frac{{\left(\mu -\frac{\sigma^{2}}{2}\right)}^{2}}{2\sigma^{2}}t\right), \end{array}$$

where we use $L^{-1}\left\{\frac{1}{s-a}\right\}\left(t\right)=e^{at}$. Applying the inverse Mellin transform and looking for the solution in the form of the convolution integral of two functions [75], $M\left\{h(x)\right\}\left(q\right)=\tilde{H}\left(q\right)$ and $M\left\{g(x)\right\}\left(q\right)=\tilde{G}\left(q\right)$,

$$M^{-1}\left\{\tilde{H}\left(q\right)\;\tilde{G}\left(q\right)\right\}\left(x\right)=\int_{0}^{\infty }h\left(r\right)\;g\left(x/r\right)\frac{dr}{r},$$

we obtain the solution of the Fokker-Planck equation for GBM

(A3) $$\begin{array}{cc} f(x,t) & =\int_{0}^{\infty }\delta \left(r-x_{0}\right)\times \frac{\exp\left(-\frac{{\left[\log\frac{x}{r}-\left(\mu -\frac{\sigma^{2}}{2}\right)t\right]}^{2}}{2\sigma^{2}t}\right)}{\left(x/r\right)\sqrt{2\pi \sigma^{2}t}}\frac{dr}{r}=\frac{1}{x\sqrt{2\pi \sigma^{2}t}}\times \exp\left(-\frac{{\left[\log\frac{x}{x_{0}}-\left(\mu -\frac{\sigma^{2}}{2}\right)t\right]}^{2}}{2\sigma^{2}t}\right). \end{array}$$

Here we used that

$$h\left(x\right)=M^{-1}\left\{x_{0}^{q-1}\right\}\left(x\right)=\delta \left(x-x_{0}\right)$$

and

$$\begin{array}{cc} g(x) & =M^{-1}\left\{\exp\left(\frac{\sigma^{2}}{2}{\left[q+\frac{1}{2}\left(\frac{2\mu }{\sigma^{2}}-3\right)\right]}^{2}t-\frac{{\left(\mu -\frac{\sigma^{2}}{2}\right)}^{2}}{2\sigma^{2}}t\right)\right\}\left(x\right)\\ & =\frac{1}{x\sqrt{2\pi \sigma^{2}t}}\times \exp\left(-\frac{{\left[\log x-\left(\mu -\frac{\sigma^{2}}{2}\right)t\right]}^{2}}{2\sigma^{2}t}\right). \end{array}$$

We used the properties of the inverse Mellin transform [75], $M^{-1}\left\{f(q+a)\right\}\left(x\right)=x^{a}M^{-1}\left\{f(q)\right\}$ and

$$M^{-1}\left\{\exp\left(\alpha q^{2}\right)\right\}\left(x\right)=\frac{1}{\sqrt{4\pi \alpha }}\;e^{-\frac{x^{2}}{4\alpha }}.$$

Therefore, from the solution (A3) we conclude that the solution of the Fokker-Planck equation is a log-normal distribution.

The *n*th moment $\langle x^{n}\left(t\right)\rangle =\int_{0}^{\infty }x^{n}P\left(x,t\right)\;dx$ of the solution of Equation (3) can be obtained by multiplying the both sides of the equation with $x^{n}$ and integration over *x*. Thus, one has

(A4) $$\begin{array}{c} \frac{\partial }{\partial t}\langle x^{n}\left(t\right)\rangle =\left[\frac{\sigma^{2}}{2}n\left(n-1\right)+\mu \;n\right]\langle x^{n}\left(t\right)\rangle , \end{array}$$

from where the *n*th moment becomes

(A5) $$\begin{array}{c} \langle x^{n}\left(t\right)\rangle =\langle x^{n}\left(0\right)\rangle \;e^{\left(\sigma^{2}n\left(n-1\right)/2+\mu \;n\right)t}. \end{array}$$

For n=0 one observes that the solution of the Fokker-Planck equation for GBM is normalised, i.e., $\langle x^{0}\left(t\right)\rangle =1$. The mean value (n=1) and the MSD have exponential dependence on time, $\langle x\left(t\right)\rangle =\langle x\left(0\right)\rangle \;e^{\mu \;t}$ and $\langle x^{2}\left(t\right)\rangle =\langle x^{2}\left(0\right)\rangle \;e^{(\sigma^{2}+2\;\mu )t}$, respectively, and thus, the variance becomes

(A6) $$\begin{array}{c} \langle x^{2}\left(t\right)\rangle -{\langle x(t)\rangle }^{2}=\langle x^{2}\left(0\right)\rangle \;e^{2\mu t}\left(e^{\sigma^{2}t}-1\right). \end{array}$$

The log-moments $\langle \log^{n}x\rangle =\int_{0}^{\infty }\log^{n}xP\left(x,t\right)\;dx$ can be obtained by multiplying the both sides of Equation (3) with $\log^{n}x$ and integration over *x*. Therefore, one finds the following equation (see Ref. [45] for details)

(A7) $$\begin{array}{c} \frac{\partial }{\partial t}\langle \log^{n}x(t)\rangle =\left(\mu -\frac{\sigma^{2}}{2}\right)n\;\langle \log^{n-1}x(t)\rangle +\frac{\sigma^{2}}{2}n\left(n-1\right)\langle \log^{n-2}x(t)\rangle . \end{array}$$

From here it follows that $\frac{\partial }{\partial t}\langle \log^{0}x(t)\rangle =0$, i.e., $\langle \log^{0}x(t)\rangle =\langle \log^{0}x(0)\rangle =1$. The case n=1 yields the mean value of the logarithm of x(t),

(A8) $$\begin{array}{c} \frac{\partial }{\partial t}\langle \log x(t)\rangle =\left(\mu -\frac{\sigma^{2}}{2}\right)\underset{=1}{\underset{︸}{\langle \log^{0}x(t)\rangle }} \end{array}$$

i.e.,

(A9) $$\begin{array}{c} \langle \log x(t)\rangle =\langle \log x(0)\rangle +\left(\mu -\frac{\sigma^{2}}{2}\right)t. \end{array}$$

For n=2 we obtain the second log-moment

(A10) $$\begin{array}{cc} \; & \frac{\partial }{\partial t}\langle \log^{2}x(t)\rangle =2\left(\mu -\frac{\sigma^{2}}{2}\right)\langle \log x(t)\rangle +\sigma^{2}\langle \log^{0}x(t)\rangle \end{array}$$

which is given by

(A11) $$\begin{array}{c} \langle \log^{2}x(t)\rangle =\langle \log^{2}x(0)\rangle +{\left(\mu -\frac{\sigma^{2}}{2}\right)}^{2}t^{2}+2\left(\mu -\frac{\sigma^{2}}{2}\right)\langle \log x(0)\rangle t+\sigma^{2}t. \end{array}$$

Therefore, for the log-variance one finds linear dependence on time

(A12) $$\begin{array}{c} \langle \log^{2}x(t)\rangle -{\langle \log x(t)\rangle }^{2}=\sigma^{2}t. \end{array}$$

## Appendix B. Derivation of the Fokker-Planck Equation for gGBM from CTRW Theory

We use the approach given in Refs. [58,76,77]. Let us consider a CTRW for a particle at position $x_{i}$ which can move right to the position $x_{i+1}=h\;x_{i}$ or to left at position $x_{i-1}=\frac{1}{h}\;x_{i}$, h>0. For the CTRW on a geometric lattice we use h=1+u, and at the end we will find the diffusion limit u→0. The probability density function (PDF) for the particle to jump to right is $p_{r}\left(x,t\right)$, and for jump to left $p_{l}\left(x,t\right)$. The total probability is $p_{r}\left(x,t\right)+p_{l}\left(x,t\right)=1$.

We consider a multiplicative jump length PDF on a geometric lattice [58],

$$\lambda \left(x_{i},t,x_{j}\right)=p_{r}\left(x_{j},t\right)\;\delta \left(x_{i}-\left[1+u\right]\;x_{j}\right)+p_{l}\left(x_{j},t\right)\;\delta \left(x_{i}-x_{j}/\left[1+u\right]\right),$$

and a waiting time PDF ψ(t), related to the survival probability by

$$\phi \left(t\right)=1-\int_{0}^{t}\psi \left(t^{\prime }\right)\;dt^{\prime },\;i.e.,\;\hat{\phi }\left(s\right)=\frac{1-\hat{\psi }\left(s\right)}{s}.$$

By substitution in the master equation [58]

(A13) $$\begin{array}{c} \frac{\partial }{\partial t}\rho \left(x_{i},t\right)=\sum_{j}\lambda \left(x_{i},t,x_{j}\right)\int_{0}^{t}K\left(t-t^{\prime }\right)\;\rho \left(x_{j},t^{\prime }\right)\;dt^{\prime }-\int_{0}^{t}K\left(t-t^{\prime }\right)\;\rho \left(x_{i},t^{\prime }\right)\;dt^{\prime }, \end{array}$$

where $K\left(t\right)=L^{-1}\left[\hat{\psi }\left(s\right)/\hat{\phi }\left(s\right)\right]$, one finds

(A14) $$\begin{array}{cc} \frac{\partial }{\partial t}\rho \left(x_{i},t\right) & =p_{r}\left(\frac{x_{i}}{1+u},t\right)\int_{0}^{t}K\left(t-t^{\prime }\right)\;\rho \left(\frac{x_{i}}{1+u},t^{\prime }\right)\;dt^{\prime }\\ & +p_{l}\left(\left[1+u\right]x_{i},t\right)\int_{0}^{t}K\left(t-t^{\prime }\right)\;\rho \left(\left[1+u\right]x_{i},t^{\prime }\right)\;dt^{\prime }-\int_{0}^{t}K\left(t-t^{\prime }\right)\;\rho \left(x_{i},t^{\prime }\right)\;dt^{\prime }. \end{array}$$

We consider generalised waiting time PDF, which in the Laplace space has the form [61,78]

$$\hat{\psi }\left(s\right)=\frac{1}{1+\tau_{\eta }/\hat{\eta }\left(s\right)},$$

where $\tau_{\eta }$ is a time parameter, which depends on η(t). Therefore,

$$\hat{\phi }\left(s\right)=\frac{\tau_{\eta }/\hat{\eta }\left(s\right)}{s\left(1+\tau_{\eta }/\hat{\eta }\left(s\right)\right)},$$

and

$$\hat{K}\left(s\right)=\frac{1}{\tau_{\eta }}\;s\times \hat{\eta }\left(s\right),$$

from where we find that $\int_{0}^{t}K\left(t-t^{\prime }\right)\;f\left(t^{\prime }\right)\;dt^{\prime }\to \frac{1}{\tau_{\eta }}\frac{d}{dt}\int_{0}^{t}\eta \left(t-t^{\prime }\right)\;f\left(t^{\prime }\right)\;dt^{\prime }$. From Equation (A14) then we obtain

(A15) $$\begin{array}{cc} \frac{\partial }{\partial t}\rho \left(x_{i},t\right) & =\frac{1}{\tau_{\eta }}\;p_{r}\left(\frac{x_{i}}{1+u},t^{\prime }\right)\frac{\partial }{\partial t}\int_{0}^{t}\eta \left(t-t^{\prime }\right)\;\rho \left(\frac{x_{i}}{1+u},t^{\prime }\right)\;dt^{\prime }\\ & +\frac{1}{\tau_{\eta }}\;p_{l}\left(\left[1+u\right]x_{i},t^{\prime }\right)\frac{\partial }{\partial t}\int_{0}^{t}\eta \left(t-t^{\prime }\right)\;\rho \left(\left[1+u\right]x_{i},t^{\prime }\right)\;dt^{\prime }-\frac{1}{\tau_{\eta }}\;\frac{\partial }{\partial t}\int_{0}^{t}\eta \left(t-t^{\prime }\right)\;\rho \left(x_{i},t^{\prime }\right)\;dt^{\prime }. \end{array}$$

Let us now consider the diffusion limit (u→0 and $\tau_{\eta }\to 0$) of Equation (A15). From the normalisation condition of the PDF ρ(x,t) given by $\sum_{i}\rho \left(x_{i},t\right)=1$ and by using position-dependent lattice spacing $\Delta x_{i}=u\;x_{i}$, one finds $\sum_{i}\frac{\rho_{u}\left(x_{i},t\right)}{u\;x_{i}}\Delta x_{i}=1$, such that $\lim_{\Delta x_{i}\to 0}\sum_{i}\left(\frac{\rho_{u}\left(x_{i},t\right)}{u\;x_{i}}\right)\Delta x_{i}=1$ [58]. By defining the function $P_{u}\left(x,t\right)=\rho_{u}\left(x,t\right)/\left[u\;x\right]$, one concludes that $P\left(x,t\right)=\lim_{u\to 0}P_{u}\left(x,t\right)$ is normalised, i.e., $\int_{0}^{\infty }P\left(x,t\right)\;dx=1$. By introducing $B_{u}\left(x_{i},t\right)=p_{r}\left(x_{i},t\right)-p_{l}\left(x_{i},t\right)$ and $b_{0}\left(x,t\right)=\lim_{u\to 0}\frac{\partial }{\partial u}B_{u}\left(x,t\right)$, in the diffusion limit u→0 and $\tau_{\eta }\to 0$, where we assume that $B_{0}\left(x,t\right)=\lim_{u\to 0}B_{u}\left(x,t\right)=0$ [58], we arrive to the following Fokker-Planck equation

(A16) $$\begin{array}{c} \frac{\partial }{\partial t}P\left(x,t\right)=D\frac{\partial }{\partial t}\int_{0}^{t}\eta \left(t-t^{\prime }\right)\frac{\partial }{\partial x}\left(x^{2}\frac{\partial }{\partial x}-\frac{1}{k_{B}T}x^{2}\;F\left(x\right)\right)P\left(x,t^{\prime }\right)\;dt^{\prime }, \end{array}$$

where $D=\lim_{u,\tau_{\eta }\to 0}u^{2}/\left[2\;\tau_{\eta }\right]$, $F\left(x\right)=-V^{\prime }\left(x\right)=k_{B}T\;\left[2\;b_{0}\left(x\right)-1\right]/x$, and $P\left(x,t\right)\propto \exp\left(-\frac{V(x)}{k_{B}T}\right)$ is obtained from the long time steady state Boltzmann distribution [58]. For a logarithmic potential $V\left(x\right)=v\;k_{B}T\;\log x$, the force becomes $F\left(x\right)=-k_{B}T\;v/x$. By using $D=\sigma^{2}/2$ and v=2−μ/D the Fokker-Planck equation becomes

(A17) $$\begin{array}{c} \frac{\partial }{\partial t}P\left(x,t\right)=\frac{\partial }{\partial t}\int_{0}^{t}\eta \left(t-t^{\prime }\right)\frac{\partial }{\partial x}\left(\frac{\sigma^{2}x^{2}}{2}\frac{\partial }{\partial x}+\left[\sigma^{2}-\mu \right]x\right)P\left(x,t^{\prime }\right)\;dt^{\prime }, \end{array}$$

which can be rewritten in the form of Equation (51).

## Appendix C. General Results for nth Moment

If we multiply both sides of Equation (51) by $x^{n}$, and integrate over *x* we find the *n*th moment $\langle x^{n}\left(t\right)\rangle =\int_{0}^{\infty }x^{n}P\left(x,t\right)\;dx$,

(A18) $$\begin{array}{c} \frac{\partial }{\partial t}\langle x^{n}\left(t\right)\rangle =\left[\frac{\sigma^{2}}{2}n\left(n-1\right)+\mu \;n\right]\mu \frac{d}{dt}\int_{0}^{t}\eta \left(t-t^{\prime }\right)\langle x^{n}\left(t^{\prime }\right)\rangle \;dt^{\prime }, \end{array}$$

from where in the Laplace space it reads

(A19) $$\begin{array}{c} \langle {\hat{x}}^{n}\left(s\right)\rangle =\frac{s^{-1}}{1-\hat{\eta }\left(s\right)\left[\frac{\sigma^{2}}{2}n\left(n-1\right)+\mu \;n\right]}\langle x^{n}\left(0\right)\rangle . \end{array}$$

From this result we obtain the normalisation condition, $\frac{\partial }{\partial t}\langle x^{0}\left(t\right)\rangle =0$, i.e., $\langle x^{0}\left(t\right)\rangle =\langle x^{0}\left(0\right)\rangle =1$. For n=1, we find the equation for the mean value

(A20) $$\begin{array}{c} \frac{\partial }{\partial t}\langle x(t)\rangle =\mu \frac{d}{dt}\int_{0}^{t}\eta \left(t-t^{\prime }\right)\langle x(t^{\prime })\rangle \;dt^{\prime }, \end{array}$$

and its Laplace pair

(A21) $$\begin{array}{c} \langle \hat{x}\left(s\right)\rangle =\frac{s^{-1}}{1-\mu \hat{\eta }\left(s\right)}\langle x(0)\rangle . \end{array}$$

In terms of the memory kernel γ(t), Equation (A21) reads

(A22) $$\begin{array}{c} \langle \hat{x}\left(s\right)\rangle =\frac{\hat{\gamma }\left(s\right)}{s\hat{\gamma }\left(s\right)-\mu }\langle x(0)\rangle . \end{array}$$

We note that for the standard case with η(t)=1 ($\hat{\eta }\left(s\right)=1/s$) we recover the previously obtained results for the GBM. For n=2 we obtain the equation for the second moment, or the MSD,

(A23) $$\begin{array}{c} \frac{\partial }{\partial t}\langle x^{2}\left(t\right)\rangle =\left(\sigma^{2}+2\mu \right)\frac{d}{dt}\int_{0}^{t}\eta \left(t-t^{\prime }\right)\langle x^{2}\left(t^{\prime }\right)\rangle \;dt^{\prime }, \end{array}$$

and its Laplace pair

(A24) $$\begin{array}{c} \langle {\hat{x}}^{2}\left(s\right)\rangle =\frac{s^{-1}}{1-\left(\sigma^{2}+2\mu \right)\hat{\eta }\left(s\right)}\langle x^{2}\left(0\right)\rangle , \end{array}$$

or

(A25) $$\begin{array}{c} \langle {\hat{x}}^{2}\left(s\right)\rangle =\frac{\hat{\gamma }\left(s\right)}{s\hat{\gamma }\left(s\right)-\left(\sigma^{2}+2\mu \right)}\langle x^{2}\left(0\right)\rangle . \end{array}$$

We also calculate the log-moments $\langle \log^{n}x(t)\rangle =\int_{0}^{\infty }\log^{n}x\;P\left(x,t\right)\;dx$, which satisfy the following integral equation

(A26) $$\begin{array}{c} \frac{\partial }{\partial t}\langle \log^{n}x(t)\rangle =\frac{\partial }{\partial t}\int_{0}^{t}\eta \left(t-t^{\prime }\right)\left[\left(\mu -\frac{\sigma^{2}}{2}\right)n\;\langle \log^{n-1}x(t^{\prime })\rangle +\frac{\sigma^{2}}{2}n\left(n-1\right)\;\langle \log^{n-2}x(t^{\prime })\rangle \right]dt^{\prime }. \end{array}$$

Thus, we find $\frac{\partial }{\partial t}\langle \log^{0}x(t)\rangle =0$, i.e., $\langle \log^{0}x(t)\rangle =\langle \log^{0}x(0)\rangle =1$. The mean value (n=1) becomes

(A27) $$\begin{array}{cc} \; & \frac{\partial }{\partial t}\langle \log x(t)\rangle =\left(\mu -\frac{\sigma^{2}}{2}\right)\frac{\partial }{\partial t}\int_{0}^{t}\eta \left(t-t^{\prime }\right)\underset{=1}{\underset{︸}{\langle \log^{0}x(t^{\prime })\rangle }}\;dt^{\prime } \end{array}$$

from where it follows

(A28) $$\begin{array}{c} \langle \log x(t)\rangle =\langle \log x(0)\rangle +\left(\mu -\frac{\sigma^{2}}{2}\right)\int_{0}^{t}\eta \left(t^{\prime }\right)\;dt^{\prime }. \end{array}$$

For the expectation of the periodic log return with period Δt, we find

(A29) $$\begin{array}{cc} \frac{1}{\Delta t}\langle \log\left(x(t+\Delta t)/x(t)\right)\rangle & =\left(\mu -\frac{\sigma^{2}}{2}\right)\frac{1}{\Delta t}\int_{t}^{t+\Delta t}\eta \left(t^{\prime }\right)\;dt^{\prime }\\ & =\left(\mu -\frac{\sigma^{2}}{2}\right)\frac{I(t+\Delta t)-I(t)}{\Delta t}\underset{\Delta t\to 0}{∼}\left(\mu -\frac{\sigma^{2}}{2}\right)\eta \left(t\right), \end{array}$$

where I(t)=∫η(t)dt, i.e., $I^{\prime }\left(t\right)=\eta \left(t\right)$. Therefore, the expectation of the periodic log returns behaves as the rate of the first log-moment,

(A30) $$\begin{array}{c} \frac{1}{\Delta t}\langle \log\left(x(t+\Delta t)/x(t)\right)\rangle \underset{\Delta t\to 0}{∼}\frac{d}{dt}\langle \log x(t)\rangle . \end{array}$$

For n=2 we obtain the second log-moment

(A31) $$\begin{array}{cc} \; & \frac{\partial }{\partial t}\langle \log^{2}x(t)\rangle =\frac{\partial }{\partial t}\int_{0}^{t}\eta \left(t-t^{\prime }\right)\left[2\left(\mu -\frac{\sigma^{2}}{2}\right)\langle \log x(t^{\prime })\rangle +\sigma^{2}\;\langle \log^{0}x(t^{\prime })\rangle \right]dt^{\prime } \end{array}$$

i.e.,

(A32) $$\begin{array}{cc} \langle \log^{2}x(t)\rangle & =\langle \log^{2}x(0)\rangle +\left\{2\left(\mu -\frac{\sigma^{2}}{2}\right)\langle \log x(0)\rangle +\sigma^{2}\right\}\int_{0}^{t}\eta \left(t^{\prime }\right)\;dt^{\prime }\\ & +2{\left(\mu -\frac{\sigma^{2}}{2}\right)}^{2}\int_{0}^{t}\eta \left(t-t^{\prime }\right)\left[\int_{0}^{t^{\prime }}\eta \left(t^{\prime \prime }\right)\;dt^{\prime \prime }\right]dt^{\prime }, \end{array}$$

and the log-variance becomes

(A33) $$\begin{array}{cc} \langle \log^{2}x(t)\rangle -{\langle \log x(t)\rangle }^{2} & =\sigma^{2}\int_{0}^{t}\eta \left(t^{\prime }\right)\;dt^{\prime }\\ & +{\left(\mu -\frac{\sigma^{2}}{2}\right)}^{2}\left[2\int_{0}^{t}\eta \left(t-t^{\prime }\right)\left(\int_{0}^{t^{\prime }}\eta \left(t^{\prime \prime }\right)\;dt^{\prime \prime }\right)dt^{\prime }-{\left(\int_{0}^{t}\eta \left(t^{\prime }\right)\;dt^{\prime }\right)}^{2}\right]. \end{array}$$

Following the same procedure, we find the third (n=3) and fourth (n=4) log-moments,

(A34) $$\begin{array}{cc} \langle \log^{3}x(t)\rangle & =\langle \log^{3}x(0)\rangle +3\left\{\left(\mu -\frac{\sigma^{2}}{2}\right)\langle \log^{2}x\left(0\right)\rangle +\sigma^{2}\langle \log x(0)\rangle \right\}I_{1}\left(t\right)\\ & +6\left(\mu -\frac{\sigma^{2}}{2}\right)\left\{\left(\mu -\frac{\sigma^{2}}{2}\right)\langle \log x(0)\rangle +\sigma^{2}\right\}I_{2}\left(t\right)+6{\left(\mu -\frac{\sigma^{2}}{2}\right)}^{3}I_{3}, \end{array}$$

(A35) $$\begin{array}{cc} \langle \log^{4}x(t)\rangle & =\langle \log^{4}x(0)\rangle +2\left\{2\left(\mu -\frac{\sigma^{2}}{2}\right)\langle \log^{3}x\left(0\right)\rangle +3\sigma^{2}\langle \log^{2}x(0)\rangle \right\}I_{1}\left(t\right)\\ & +6\left\{2\left(\mu -\frac{\sigma^{2}}{2}\right)\left[\left(\mu -\frac{\sigma^{2}}{2}\right)\langle \log^{2}x(0)\rangle +2\sigma^{2}\langle \log x(0)\rangle \right]+\sigma^{4}\right\}I_{2}\left(t\right)\\ & +24{\left(\mu -\frac{\sigma^{2}}{2}\right)}^{2}\left\{\left(\mu -\frac{\sigma^{2}}{2}\right)\langle \log x(0)\rangle +3\sigma^{2}/2\right\}I_{3}\left(t\right)+24{\left(\mu -\frac{\sigma^{2}}{2}\right)}^{4}I_{4}\left(t\right), \end{array}$$

where

(A36) $$\begin{array}{cc} \; & I_{1}\left(t\right)=\int_{0}^{t}\eta \left(t_{1}\right)\;dt_{1}, \end{array}$$

(A37) $$\begin{array}{cc} & I_{2}\left(t\right)=\int_{0}^{t}\eta \left(t-t_{1}\right)\left[\int_{0}^{t_{1}}\eta \left(t_{2}\right)\;dt_{2}\right]dt_{1}, \end{array}$$

(A38) $$\begin{array}{cc} \; & I_{3}\left(t\right)=\int_{0}^{t}\eta \left(t-t_{1}\right)\left\{\int_{0}^{t_{1}}\eta \left(t_{1}-t_{2}\right)\left[\int_{0}^{t_{2}}\eta \left(t_{3}\right)\;dt_{3}\right]dt_{2}\right\}dt_{1}, \end{array}$$

(A39) $$\begin{array}{cc} \; & I_{4}\left(t\right)=\int_{0}^{t}\left(\eta \left(t-t_{1}\right)\int_{0}^{t_{1}}\eta \left(t_{1}-t_{2}\right)\left\{\int_{0}^{t_{2}}\eta \left(t_{2}-t_{3}\right)\left[\int_{0}^{t_{3}}\eta \left(t_{4}\right)\;dt_{4}\right]dt_{3}\right\}dt_{2}\right)dt_{1}. \end{array}$$

## Appendix D. Fox *H*-Function

The Fox *H*-function is defined by [79]

(A40) $$\begin{array}{c} H_{p,q}^{m,n}\left[z\left|\begin{array}{c} \left(a_{1},A_{1}\right),\cdots ,\left(a_{p},A_{p}\right)\\ \left(b_{1},B_{1}\right),\cdots ,\left(b_{q},B_{q}\right) \end{array}\right.\right]=H_{p,q}^{m,n}\left[z\left|\begin{array}{c} \left(a_{p},A_{p}\right)\\ \left(b_{q},B_{q}\right) \end{array}\right.\right]=\frac{1}{2\pi ı}\int_{\Omega }\theta \left(s\right)z^{s}\;ds, \end{array}$$

where θ(s) is given by $\theta \left(s\right)=\frac{\prod_{j=1}^{m}\Gamma \left(b_{j}-B_{j}s\right)\prod_{j=1}^{n}\Gamma \left(1-a_{j}+A_{j}s\right)}{\prod_{j=m+1}^{q}\Gamma \left(1-b_{j}+B_{j}s\right)\prod_{j=n+1}^{p}\Gamma \left(a_{j}-A_{j}s\right)}$, 0≤n≤p, 1≤m≤q, $a_{i},b_{j}\in C$, $A_{i},B_{j}\in R^{+}$, i=1,...,p, j=1,...,q. The contour Ω starting at c−ı∞ and ending at c+ı∞ separates the poles of the function $\Gamma (b_{j}+B_{j}s)$, j=1,...,m from those of the function $\Gamma (1-a_{i}-A_{i}s)$, i=1,...,n.

A special case of the Fox *H*-function is the exponential function [79],

(A41) $$\begin{array}{c} e^{-z}=H_{0,1}^{1,0}\left[z\left|\begin{array}{c} -\\ \left(0,1\right) \end{array}\right.\right]. \end{array}$$

The inverse Laplace transform of the Fox *H*-function reads [79]

(A42) $$\begin{array}{c} L^{-1}\left[s^{-\rho }\;H_{p,q}^{m,n}\left[a\;s^{\sigma }\left|\begin{array}{c} \left(a_{p},A_{p}\right)\\ \left(b_{q},B_{q}\right) \end{array}\right.\right]\right]\left(t\right)=t^{\rho -1}\;H_{p+1,q}^{m,n}\left[\frac{a}{t^{\sigma }}\left|\begin{array}{c} \left(a_{p},A_{p}\right),\left(\rho ,\sigma \right)\\ \left(b_{q},B_{q}\right) \end{array}\right.\right]. \end{array}$$

The Fox *H*-functions have the following property [79]

(A43) $$\begin{array}{c} z^{k}\;H_{p,q}^{m,n}\left[z\left|\begin{array}{c} \left(a_{p},A_{p}\right)\\ \left(b_{q},B_{q}\right) \end{array}\right.\right]=H_{p,q}^{m,n}\left[z\left|\begin{array}{c} \left(a_{p}+kA_{p},A_{p}\right)\\ \left(b_{q}+kB_{q},B_{q}\right) \end{array}\right.\right]. \end{array}$$
